# SSDBFAN: Scalable and Secure Cluster-Based Data Aggregation with Blockchain for Flying Ad Hoc Networks

**DOI:** 10.3390/s26092585

**Published:** 2026-04-22

**Authors:** Sufian Al Majmaie, Ghazal Ghajari, Niraj Prasad Bhatta, Mohamed I. Ibrahem, Fathi Amsaad

**Affiliations:** 1 Department of Computer Science and Engineering, Wright State University, Dayton, OH 45435, USA; al-majmaie.2@wright.edu (S.A.M.); ghajari.2@wright.edu (G.G.); 2 Department of Computer Engineering, University of Cincinnati, Cincinnati, OH 45219, USA; bhattand@mail.uc.edu; 3 Cyber Systems Engineering Department, Augusta University, Augusta, GA 30912, USA; mibrahem@augusta.edu

**Keywords:** FANET, blockchain, data aggregation, cryptography, optimization algorithms

## Abstract

Mobile Unmanned Aerial Vehicles (UAVs) forming Flying Ad Hoc Networks (FANETs) offer promising applications, but dynamic network structures, limited resources, and potential single points of failure create security challenges. While cluster-based data aggregation, where data is collected and combined at Cluster Heads (CHs) before transmission, improves efficiency, traditional techniques can compromise data privacy. This paper introduces SSDBFAN, a scalable and secure cluster-based data aggregation framework for Flying Ad Hoc Networks (FANETs). The proposed approach integrates the Frilled Lizard Optimization Algorithm (FLOA) for efficient cluster head selection with blockchain technology and post-quantum cryptographic techniques, including lattice-based homomorphic encryption and the Chinese Remainder Theorem, to ensure privacy-preserving data aggregation. Additionally, a hybrid online/offline signature mechanism is employed to achieve secure and efficient authentication with reduced computational overhead. The performance of the proposed framework is evaluated using NS-3 simulations under varying network sizes. Experimental results demonstrate that SSDBFAN significantly improves communication efficiency, reduces computational cost, and enhances network stability compared to existing schemes. Furthermore, scalability analysis with up to 500 UAV nodes confirms that the proposed framework effectively controls blockchain overhead, including bandwidth consumption, consensus latency, and storage requirements. Comparative evaluation with existing optimization algorithms shows that FLOA achieves superior performance in terms of cluster stability, delay, and throughput. These results validate the effectiveness of SSDBFAN as a scalable and security-aware solution for large-scale FANET environments.

## 1. Introduction

Unmanned Aerial Vehicles (UAVs), commonly known as drones, have gained widespread adoption across various wireless communication applications due to their cost efficiency, mobility, and flexibility. These airborne nodes can serve as aerial base stations, enhancing network capacity, coverage, and energy efficiency. UAVs come in different forms, including small aircraft, balloons, and drones, and can be either remotely controlled or pre-programmed for autonomous operation. Their applications span multiple domains, including military operations, surveillance, search and rescue missions, and telecommunications. However, securing communication in UAV networks poses a significant challenge due to their dynamic topology, resource constraints, and susceptibility to cyber threats.

To safeguard communication in UAV networks, encryption mechanisms, both symmetric and asymmetric, are commonly employed. A robust key management strategy is essential for their effectiveness. Prior studies have explored group key management solutions to enhance security in UAV networks. For instance, Mao et al. [1] proposed a dynamic group key management approach where the key generation center was selected based on routing information. Similarly, Semal et al. [2] introduced a certificateless group key agreement protocol, enabling drones to securely negotiate group keys. However, these approaches lacked key update mechanisms, meaning secret keys remained unchanged after initialization, making them vulnerable to long-term attacks.

Further research by Hong et al. [3] proposed a provably secure aggregate authentication scheme that enables batch verification of UAV data. Despite its security benefits, this method also failed to address key updates. A different approach was presented by Li et al. [4], who designed a mutual healing group key distribution scheme using a private blockchain. While this method centralized key management at the base station (BS), it introduced a single point of failure; if the BS were compromised, the entire communication system would be at risk. To overcome these limitations, blockchain technology has emerged as a promising solution for secure and decentralized key management [5,6]. Blockchain’s tamper-resistant structure and decentralized nature eliminate reliance on a single trusted authority, making it ideal for dynamic networks like FANETs. In a blockchain system, transactions are securely recorded and traceable, ensuring data integrity and enabling auditable key updates. These unique properties make blockchain a viable alternative to traditional key management schemes.

The proposed framework follows a hybrid trust model, where the Trusted Authority (TA) is only involved during initialization, and the Ground Control Station (GCS) operates as a semi-trusted entity. Blockchain ensures decentralized verification during runtime, thereby reducing reliance on centralized control while maintaining practical deployment feasibility. Although blockchain is seen as a simple tool for logging data, its use within the proposed SSDBFAN architecture goes far beyond simple logging. Within this work, blockchain is leveraged to allow for decentralized trust management, aggregatable data, and auditability within highly dynamic UAV environments. Unlike traditional architectures, where a single entity is responsible for trust, a blockchain-based system distributes this trust among multiple entities, effectively eliminating a single point of failure. Furthermore, this distributed approach also allows for the immutable storage of aggregated results, ensuring traceability and accountability within mission-critical UAV applications such as surveillance and disaster response. This system-level integration ensures that security is enforced at both the architectural and cryptographic levels. To overcome the drawbacks related to secure and privacy-guaranteed data aggregation and improve blockchain features, the following contributions are made in this paper.

1. A proposed technique called SSDBFAN that leverages FANET networks is implemented. It employs a CH-based approach for collecting data from members in the cluster and distributing load moderately across the network. This aids in reducing computational load on nodes, enabling seamless and efficient operation.
2. An optimal clustering mechanism based on the Frilled Lizard Optimization Algorithm (FLOA) is implemented in terms of mobility, trust, and energy consumption.
3. SSDBFAN uses an online/offline signature scheme, reducing computational costs related to data integrity operations, making it suitable for FANET devices, as it reduces processing requirements and enables smooth operation.
4. The proposed system is implemented using NS-3 simulations, performance is analyzed, and security analysis of the proposed work is carried out.
5. A detailed security analysis that shows how SSDBFAN aids in achieving data integrity, confidentiality, authentication, as well as privacy preservation, successfully using the considered threat model.

The remainder of this paper is organized as follows. Section 2 reviews the related work. Section 3 presents the system model and the proposed SSDBFAN framework. Section 4 provides the security analysis, while Section 5 discusses the performance evaluation. Finally, Section 6 concludes the paper and outlines future research directions.

## 2. Related Work

### 2.1. Blockchain for FANET Networks

Recent research has investigated the integration of blockchain technology into Flying Ad Hoc Networks (FANETs), including heterogeneous drone networks, to enhance security and management. Recent work proposed a blockchain-based Distributed Key Management System to manage the encryption keys in cluster-based approaches that can make the mining process faster and more energy-efficient [7]. Each cluster has a leading head drone that coordinates the application-related activities in the blockchain, including mining and cluster key distribution.

Additionally, a Secure Authentication System was proposed for drone-assisted smart vehicle networks where smart contracts are used to validate the authentication of drones and other entities in providing secure communication [8]. Another vital contribution is found in the low-latency security authentication within smart cities. It is based on a decentralized consensus mechanism, namely, Drone-based Delegated Proof of Stake (DDPoS) [9], which improves security for IoT devices, reduces latency, and optimizes metrics such as packet loss rate and throughput. Other research has integrated Blockchain with ML for intelligent processing of data captured by drones, including their privacy. Participating drones shared the ML model to enhance the accuracy of the analysis, while blockchain guarantees the integrity of data [10].

### 2.2. Data Aggregation and Key Management

Key management in mobile networks, especially in VNets, provides a useful starting point for FANETs due to the related challenges caused by their dynamic and distributed nature. In VNets, there often exists a three-tier architecture: a Trusted Authority (TA), RSUs, and network nodes. Central management of credentials of all vehicles is possible with TA, and RSUs serve as the intermediate nodes for secure communication between TA and vehicles [11]. However, in a centralized model, there are certain vulnerabilities, such as single points of failure. This makes the centralized model less ideal in highly dynamic FANETs, where the mobility of drones is even more unpredictable. Such limitations have driven research towards the development of decentralized blockchain-based systems for key management in which no central authority is involved.

In one blockchain-based system, through the utilization of smart contract capabilities, dynamic key management has been enabled; this allows drones to perform autonomous key management for encryption [12]. The system ensures dynamic key updates in the case of a drone joining or leaving the network, which is particularly vital in FANETs, where node mobility and changes in network topology frequently occur. Another promising approach involves dynamic key management for Vehicle Platooning in VNet, wherein vehicles travel in a coordinated group [13]. Such a concept applies to FANETs with regard to cluster key management over a set of drones operating in proximity.

Lightweight mutual-healing key distribution schemes have also been proposed where drones autonomously recover from key compromise scenarios without the need for extensive communication overhead [14]. By applying the distributed ledger technology of blockchain, this approach guarantees that the key update process is secure, traceable, and tamper-evident. This type of key management reduces energy consumption without compromising security for FANETs, which consist of resource-constrained drones. However, in comparison with VNets, FANETs present different challenges: high mobility, low computational resources, and flying drones. As a result, these factors lead to difficulty in applying conventional key management schemes as designed for terrestrial networks. The unique demands of FANETs call for further advancements in lightweight, energy-efficient key management systems.

### 2.3. Drawbacks in Existing Systems and Proposed Solutions

The particular difficulties that still exist in blockchain-based solutions used for managing heterogeneous drone networks include risks to data security, such as tampering, unauthorized access, and single-point failures, as well as scalability-related issues like network congestion and storage limitations. Furthermore, consensus algorithms like Proof of Work (PoW) result in excessive energy consumption and very low processing speed, limiting real-time applications in drones. Another serious challenge is interoperability, with compatibility between different varieties of drones and blockchain platforms.

Zhou et al. [15] proposed a dynamic group key agreement scheme utilizing blockchain and certificateless cryptography to avoid centralized key escrow and improve trust. Their system supports key updates and cluster head election via Raft consensus. Nevertheless, it lacks any optimization method for secure cluster formation and does not address privacy-preserving data aggregation, relying solely on certificateless methods without homomorphic or post-quantum capabilities. Hong et al. [16] extended the key agreement paradigm to support high-mobility UAV environments using a decentralized blockchain ledger. Although their scheme enables lost key recovery and fair consensus, it similarly omits support for cryptographic data aggregation and fails to employ clustering or lightweight optimization techniques for efficient communication. In another notable work, Li et al. [4] introduced a blockchain-based mutual-healing group key distribution protocol specifically for highly dynamic UAV networks. Their model leverages a private blockchain maintained by the Ground Control Station (GCS) to support key recovery and node management.

Hafeez et al. [17] proposed a blockchain-enhanced UAV network for disaster recovery featuring a hybrid consensus protocol (DPoS-PBFT) and decentralized flocking coordination. Their solution achieves scalability and resilience but does not incorporate cryptographic signatures or privacy-preserving aggregation methods, nor does it support post-quantum cryptographic primitives. More recent studies have looked at lightweight authentication and key agreement methods for UAV networks. However, there are still several limitations when it comes to secure and scalable data aggregation in FANET environments. Yuwen et al. [18] proposed a blockchain-assisted lightweight authentication method using covert communication to improve security against eavesdropping and impersonation attacks. While their approach lowers authentication overhead and enhances scalability at the protocol level, it does not support clustering, privacy-preserving data aggregation, or advanced cryptographic techniques like homomorphic encryption. This limits its usefulness in data-intensive FANET scenarios.

Liu et al. [19] introduced EUAV, a blockchain-based two-factor anonymous authentication and key agreement protocol aimed at increasing user anonymity and resisting key compromise attacks. Although EUAV provides better authentication security and efficiency through elliptic-curve cryptography and blockchain integration, it mainly focuses on access authentication. It does not tackle secure data aggregation, clustering optimization, or post-quantum resilience, which are critical for large-scale FANET deployments. Similarly, Sen et al. [20] presented a PUF-based authentication framework for UAV flying ad hoc networks, which aims to lower key storage overhead and improve resistance to physical attacks. Despite its lightweight design and stronger authentication, the scheme lacks blockchain integration, digital signature support, privacy-preserving aggregation, and clear key update methods. This makes it unsuitable for decentralized and data-driven FANET applications.

To address the above limitations, our proposed SSDBFAN framework introduces a Frilled Lizard Optimization Algorithm (FLOA) for efficient cluster head selection, enabling scalable, mobility-aware, and energy-efficient clustering. It leverages lattice-based Fully Homomorphic Encryption (FHE) and Chinese Remainder Theorem (CRT) for privacy-preserving data aggregation and introduces hybrid digital signatures (combining BLS-SF, HSSP, and Online/Offline modes) to ensure secure communication under post-quantum constraints. Furthermore, a blockchain ledger integrated with Merkle trees and dynamic key updates supports decentralized trust without reliance on centralized nodes, making SSDBFAN a comprehensive and scalable solution for secure UAV data aggregation in Flying Ad Hoc Networks (FANETs). Table 1 presents a qualitative comparison of existing approaches based on key design features, advantages, and limitations. It is intended for conceptual comparison rather than quantitative performance evaluation.

## 3. Proposed SSDBFAN Framework

### 3.1. System Model

The proposed FANET structure involves four main components: Trusted Authority (TA), Ground Control Stations (GCSs), Cluster Heads (CHs), and Cluster Members (CMs). The TA is the entity that initiates the system in a secure manner by distributing keys and parameters to all nodes, after which it is disconnected from the network. GCSs are semi-offline controllers that perform network setup, handle system configuration, and distribute key material securely at initialization time.

CHs are trust-, energy-, and connectivity-based selected nodes that form local clusters, handle intra-cluster communications, and aggregate encrypted messages from nodes of the local cluster (CMs). CMs are resource-constrained UAVs that collect sensing data from the environment, encrypt and check for integrity, and send the protected data to the assigned CHs for further analysis. The SSDBFAN architecture has been designed in a multi-layered structure, where each component plays a vital role in meeting the security and performance requirements. In this structure, the Trusted Authority’s role is limited to initialization and key distribution, following which it is excluded to prevent centralization attacks. On the other hand, the Cluster Heads are responsible for handling computationally expensive operations like aggregation and partial verification. This offloads the UAV nodes, which are resource-constrained. Finally, the Ground Control Stations are considered to be semi-trusted parties. The blockchain component has been designed to provide a separate trust layer, ensuring the immutability of the data, decentralized verification, and secure key update tracking. In this structure, the homomorphic encryption provides end-to-end confidentiality, signatures provide integrity and authenticity, and CRT provides efficient reconstruction of aggregated data. Each component in this structure has been designed to avoid bottlenecks and to reduce inter-component dependencies.

### 3.2. Workflow of SSDBFAN

SSDBFAN operates in two primary phases: cluster selection and data aggregation. The Frilled Lizard Optimization Algorithm (FLOA) is used to dynamically create the clusters and choose the best Cluster Heads (CHs) based on trust levels, mobility patterns, and remaining energy levels in the first stage. Then, a multi-step data aggregation is carried out for security and efficiency purposes.

The system is initialized with parameters $\left(s,s_{1}\right)$, public keys $\Psi_{pub}$, and master key $M_{key}$. Secure registration is done at each node by creating a public key $P_{i}$ and a registration tuple $\left(\sigma_{i},\tau_{i}\right)$ utilizing blinding factors and random numbers. Then, sign-off generates offline signatures $\Sigma_{i}^{offline}$, state info $SA_{t}$, and an online verification key $VK_{online}$. During report generation, encrypted data reports $er_{i}$ are generated using public keys and messages, along with online signatures $\Sigma_{i}^{online}$. Then, they are verified and aggregated by CHs to form Ω, and an aggregation signature $\Sigma_{Aggr}$ is generated. (GCSs) authenticate the aggregation using $VK_{Aggr}$, and on success, recover the original report using the recovery function $Recov_{Aggr}\left(c\right)$.

**Figure 1:**
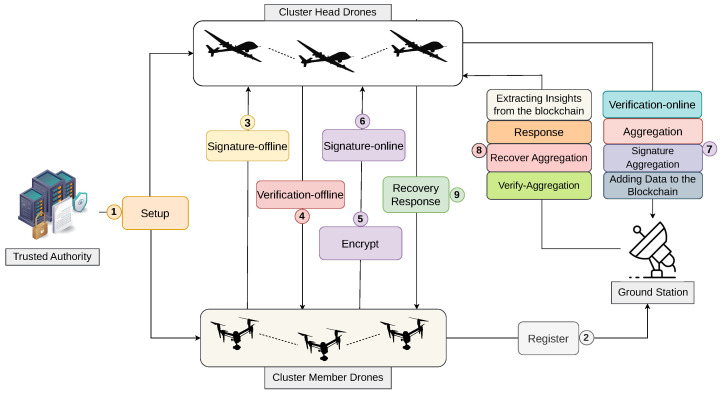
Overall architecture of SSDBFAN.

Then, encrypted responses $\left({\tilde{er}}_{1},{\tilde{er}}_{2},{\tilde{er}}_{3}\right)$ are generated at GCSs, which are then decrypted by authorized nodes using the reveal recovery function $Recov_{res}$. Lastly, aggregated data $AGP_{x}$ is added to the blockchain and GCSs mine insights using the Chinese Remainder Theorem (CRT) to obtain the complete dataset $U=\{U_{1},U_{2},\ldots ,U_{k}\}$, facilitating secure and privacy-preserving usage of the data throughout FANET components. Figure 1 illustrates the SSDBFAN architecture, showing registration, secure data aggregation, and blockchain-based communication among FANET entities.

### 3.3. Threat Model

Data aggregation requires strong security features to ensure the privacy of the users. In the proposed security model, the Trusted Authority (TA) is only assumed to be fully trusted during the initialization phase, in which the system parameters are created, and the keys are exchanged among the entities in the network. In the subsequent phases, the TA is not involved in the network operations, thus, eliminating the need to rely on a centralized entity. The Ground Control Station (GCS) is considered a semi-trusted party, which validates the data aggregation process and interacts with the blockchain network but does not access the data in plain format. This is a more realistic assumption, considering the honest-but-curious nature of the entities in the network. On the other hand, the Cluster Head (CH) is also considered a semi-trusted party, which acts honestly in the data aggregation process but is curious about the data, implying that the Cluster Head will not alter the data but will try to extract the private information during the data aggregation process. In addition, an external attacker, represented as A, is also present in the communication network, which aims to compromise the network operations by violating the integrity of the data and extracting the private information using active attacks, including eavesdropping, unauthorized access, or sending fake data. In the proposed security model, the blockchain technology is integrated to distribute the trust among the entities in the network, thus, eliminating the need to rely on a single entity in the network, which is in line with the decentralized security concept. Hence, the security features must ensure the confidentiality, authentication, integrity, and privacy of the data in the network, considering both internal and external attacks.

To avoid unnecessary complexities and achieve a coherent system design, all components of the proposed SSDBFAN framework have been carefully selected to address a particular limitation in UAV network security and scalability.

- The proposed FLOA-based clustering mechanism is responsible for efficient cluster head selection for enhanced energy balance, stability, and scalability.
- Lattice-based Fully Homomorphic Encryption (FHE) is proposed for achieving data confidentiality during aggregation without compromising raw data.
- The proposed online/offline signature scheme is efficient for achieving node authentication with low computational overhead, making it suitable for UAV nodes.
- The proposed Chinese Remainder Theorem (CRT) is efficient for achieving data aggregation efficiency and reducing computational complexities during reconstruction.
- Blockchain technology is proposed for achieving data storage security and verification.

Each component of the proposed SSDBFAN framework is independent and contributes to achieving a particular security and scalability requirement. The proposed components have been selected to achieve a cohesive system design without compromising security, efficiency, and scalability.

### 3.4. Design Goals

Based on the system and threat models, the proposed scheme involves 4 primary design objectives:

- **Protecting Confidential Information:** The sensitive data of nodes will be encrypted once it leaves their devices to maintain privacy. Furthermore, only aggregated results will be accessible to internal adversaries, such as CH, and individual data will not be accessible.
- **Verification of Identity and Data Integrity:** Nodes which are part of SSDBFAN system must be authorized by the GCSs. Adversaries will not be able to manipulate data during transmission, and any unauthorized modifications will be detected by the GCSs and CH.
- **Efficient Processing:** The scheme aims to simplify complex operations to reduce the computational burden on mobile nodes. In addition, efficient communication is essential for handling frequent aggregation requests.
- **Adaptable to Various Networks:** The SSDBFAN scheme can be easily implemented in other network settings such as smart grids and node sensing systems. Its lightweight nature also makes it easy to integrate into new systems.

### 3.5. CH Selection

The best CH in the network is selected using the FLOA which takes into account various parameters such as energy consumption, trust level and mobility [21]. The process of cluster selection is as follows.

#### Trust Calculation

Trust is a critical factor involved in determining the node to be selected as the CH for data transmission. The trust level (T) of a node is assessed based on two main components: Its (i) Experience (E) and (ii) Reputation (R). The symbols and notations used in calculating the trust score are outlined in Table 2. Each node’s trust value is computed using Equations (1) and (2), where the weights assigned to experience and reputation, denoted as $w_{R}$ and $w_{E}$ respectively satisfy the condition $w_{R}+w_{E}=1$. Both parameters are given equal importance when determining the final trust score.

(1) $$\mathrm{Trust}=w_{E}\times E+w_{R}\times R,$$

(2) $$\mathrm{Trust}=\frac{1}{2}\times E+\frac{1}{2}\times R.$$

In the current study, the weights $\omega_{E}$, $\omega_{R}$ are set to 0.5 in order to ensure the balanced contribution of experience and reputation, but the model is not simply an averaging approach, as the two components are dynamically updated based on the nodes’ behavior and interaction history.

The notations used for our trust calculation are defined as follows: $E_{o}$, $E_{\min}=0$ and $E_{\max}$ represent the experience level of a node that has recently joined, least and maximum experience of a node respectively. Experience is normalized within the range [0, 1]. $R_{\min}=0$ represents minimum experience of a node. α and β denote the feedback score after successful and unsuccessful transactions. $E_{t}$ and $E_{t+1}$ denote the current and updated experience levels of a node. $S_{T}$ and $U_{T}$ denote the number of successful and unsuccessful transactions. Further, $S_{T({\mathrm{CLS}}_{1}-{\mathrm{CLS}}_{2})}$ and $U_{T({\mathrm{CLS}}_{1}-{\mathrm{CLS}}_{2})}$ denote successful and unsuccessful transactions between clusters ${\mathrm{CLS}}_{1}$ and ${\mathrm{CLS}}_{2}$ respectively.

(1) Experience Level

A node’s experience level (*E*) is determined based on two factors: positive experience ($E_{\mathrm{pos}}$) and negative experience ($E_{\mathrm{neg}}$). Positive transactions ($T_{\mathrm{pos}}$) contribute to increase in a node’s trust value, while negative transactions ($T_{\mathrm{neg}}$) reduce it. *E* is dynamically updated after each transaction, reflecting the node’s ongoing performance and behavior.

**Positive Experience:** Node *n* functions as CH for a transmission. After successful transaction, $E_{\mathrm{pos}}$ follows Equation (3).

(3) $$E_{t+1}=E_{t}+\alpha \Delta ,$$

where Δ is defined in Equation (4) with η as the value to normalize experience value in the range [0, 1].

(4) $$\Delta =\eta \times (1-E_{t}).$$

where η is a normalization factor (0<η≤1) that controls the rate of increase of the experience value. It ensures that the experience remains within the range [0,1] and prevents abrupt changes in trust values. In this work, η is set to a small constant (e.g., η=0.1) to achieve stable and gradual updates.

**Negative Experience:** Node *n* functions as CH for a transmission. After unsuccessful transmission, $E_{\mathrm{neg}}$ follows Equation (5).

(5) $$E_{t+1}=\max\left(E_{\min},E_{t}-\beta \right).$$

(2) Reputation Factor

The reputation factor (*R*) is derived by combining both values of intra and inter-cluster trust. During a transmission, the communicating nodes, including the CH and node (*n*), may either belong to the same cluster or different clusters. When the nodes are part of the same cluster, the trust established is referred to as direct trust ($T_{D}$). Conversely, if the nodes are in separate clusters, the resulting trust is considered indirect trust ($T_{ID}$). The direct trust ($T_{D}$) is computed using Equation (6).

(6) $$T_{D}=\left\{\begin{array}{cc} \max(R_{\min},\frac{S_{T}-U_{T}}{S_{T}+U_{T}}), & \mathrm{if}\;S_{T},U_{T}\neq 0\\ 0, & \mathrm{otherwise} \end{array}\right..$$

For example, if node *i* belonging to cluster $C_{1}$ chooses node *j* as CH of cluster $C_{2}$, then $T_{ID}$ of CH is determined as shown in Equation (7).

(7) $$T_{ID}=\left\{\begin{array}{cc} \max(R_{\min},\frac{S_{T(C_{1}-C_{2})}-U_{T(C_{1}-C_{2})}}{S_{T(C_{1}-C_{2})}+U_{T(C_{1}-C_{2})}}), & \mathrm{if}\;S_{T(C_{1}-C_{2})},U_{T(C_{1}-C_{2})}\neq 0\\ 0, & \mathrm{otherwise} \end{array}\right..$$

(3) Mobility Factor

Mobility factor used in clustering is determined based on Relative velocity Average (RelVA), the Distance average (DA), and the Stability of Link (SL).

(8) $$MV_{i}=\frac{SL_{i}\left(t\right)}{NVN_{i}}+\frac{RelVA_{i}}{{\mathrm{maxv}}_{i}}+\frac{DA_{i}}{\max D_{i}},$$

where, NVN is the number of neighbors per vehicle that is based on vehicle (*i*) degree, $\max_{i}$ the maximum distance of *i*, and $\max D_{i}$ maximum speed of *i*. The average velocity of *i* is given by,

(9) $$RelVA_{i}=\frac{1}{NVN_{i}}\sum_{\begin{array}{c} j=1\\ j\neq i \end{array}}^{NVN_{i}}\left|v_{i}-v_{j}\right|,$$

where, $v_{i}$ represents velocity of *i*. The lower the value of RelVA for *i* (node), the more stable the vehicle. $M_{\mathrm{dist}}$ represents the mean distance between *i* and its neighbors and is calculated as shown below.

(10) $$DM_{{\mathrm{dist}}_{i}}=\frac{1}{NVN_{i}}\sum_{\begin{array}{c} j=1\\ j\neq i \end{array}}^{NVN_{i}}\sqrt{{\left(a_{i}-a_{j}\right)}^{2}+{\left(b_{i}-b_{j}\right)}^{2}},$$

where, $\left(a_{i},b_{i}\right)$ denotes the respective coordinates of *i* and its neighbors. Further, it is assumed that vehicles closer to the center have a minimum $M_{\mathrm{dist}}$. Stability of Link (SL) represents the stability of the communication link between *i* and its neighbors, which is dependent on $M_{\mathrm{dist}}$. $SL_{i}\left(t\right)$ of *i* over time $t=(t_{2}-t_{1})$ is computed as below, where, $M_{{\mathrm{dist}}_{i}}\left(t_{1}\right)$ and $M_{{\mathrm{dist}}_{i}}\left(t_{2}\right)$ represent $M_{\mathrm{dist}}$ at time $t_{1}$ and $t_{2}$ of *i* respectively.

(11) $$SL_{i}\left(t\right)=\left|M_{{\mathrm{dist}}_{i}}\left(t_{1}\right)-M_{{\mathrm{dist}}_{i}}\left(t_{2}\right)\right|.$$

(4) Energy Parameter

Owing to the high-speed mobility of nodes, CHs need to periodically broadcast HELLO messages to update the clustering structure and monitor link connection status among their neighbors. In addition, CHs play a crucial role in data aggregation, communication, and coordination within their respective clusters. Nodes with higher energy reserves are better equipped to handle these responsibilities, ensuring more balanced energy consumption across the network. Hence, the remaining energy during CH election is considered. For node *i*, the remaining energy $E^{re}\left(i\right)$ can be calculated by:

(12) $$E^{re}\left(i\right)=E_{i0}-E_{ic},$$

where $E_{i0}$ and $E_{ic}$ represent initial and consumed energy of transmitting and receiving packets respectively. As the 2-hop neighbor range is considered, normalized energy for 2-hop range is determined as shown below:

(13) $$\phi_{i}=\frac{E^{re}\left(i\right)}{\max[E_{N_{2}}^{re}\left(i\right)]},$$

where, $\phi_{i}$ denotes normalized EF and $E_{N_{2}}^{re}\left(i\right)$ represents residual energy of the 2-hop node (i).

(5) Fitness Function

In the dynamic mobile environment, it is essential to design objective functions tailored to the specific requirements of FANETs. A well-rounded objective function should be capable of efficiently addressing diverse features and operational demands. Rather than relying on separate objective functions for applications with different Quality of Service (QoS) and adjacent nodes, this approach adopts a weighted parameter model that accommodates all data flow types. The fitness function integrates key factors like mobility, energy efficiency, and trust [22]. It represents the weight of the *j*-th node as a potential forwarding candidate from the *i*-th node. This fitness function, defined in Equation (14), is used as input to FLOA, which determines the optimal forwarding node depending on decisions made by FLOA:

(14) $$F_{j}=\delta_{1}\times {\mathrm{Trust}}_{i}+\delta_{2}\times MV_{i}+\delta_{3}\times \phi_{i},$$

where, $\delta_{1}$, $\delta_{2}$, $\delta_{3}$ are relative coefficients used for routing, $\delta_{1}+\delta_{2}+\delta_{3}=1$, $MV_{i}$ represents the mobility factor of *i*, ${\mathrm{Trust}}_{i}$ signifies trust assessment of *i*, and $\phi_{i}$ represents normalized energy consumption of *i*.

### 3.6. Frilled Lizard Optimization Algorithm (FLOA)

This section outlines the conceptual foundation and inspiration behind the development of the FLOA [21]. Following this, the algorithm’s implementation process is mathematically formulated to address a range of optimization problems.

#### 3.6.1. Algorithm Initialization

The proposed FLOA is a nature-inspired metaheuristic that models the behavior of FLs. It identifies near-optimal solutions for optimization problems by using the exploratory abilities of the members in Solution Space (SS). Every FL represents a Candidate Solution (CS), assigning values to decision variables depending on a specific location in the search space. Mathematically, each FL is represented as a vector with the entire group forming the FLOA population represented as a matrix in Equation (15). Initial locations of FLs within the SS are determined by random initialization as shown in Equation (16).

(15) $$X={\left[\begin{array}{c} X_{1}\\ \vdots \\ X_{i}\\ \vdots \\ X_{N} \end{array}\right]}_{N\times m}={\left[\begin{array}{ccccc} x_{1}^{1} & \ldots & x_{1}^{d} & \ldots & x_{1}^{m}\\ \vdots & \ddots & \vdots & \ldots & \vdots \\ x_{i}^{1} & \ldots & x_{i}^{d} & \ldots & x_{i}^{m}\\ \vdots & \ldots & \vdots & \ddots & \vdots \\ x_{N}^{1} & \ldots & x_{N}^{d} & \ldots & x_{N}^{m} \end{array}\right]}_{N\times m},$$

(16) $$x_{i,d}=lb_{d}+r\cdot \left(ub_{d}-lb_{d}\right).$$

‘X’ denotes FLOA population matrix with ‘$X_{i}$’ denoting *i*-th FL (CS). $x_{i}^{d}$ refers to *d*-th dimension of *i*-th FL in search space (decision variable). *N* represents total number of FLs, and *m* represents number of decision variables. *r* denotes random number within limits [0, 1], while $lb_{d}$ and $ub_{d}$ define lower as well as upper bounds of *d*-th decision variable.

Since every FL is considered as a potential solution, the corresponding value of the objective function for each CS may be determined. The set of objective function values is given as a vector as presented in Equation (17).

(17) $$F={\left[\begin{array}{c} F_{1}\\ \vdots \\ F_{i}\\ \vdots \\ F_{N} \end{array}\right]}_{N\times 1}={\left[\begin{array}{c} F(X_{1})\\ \vdots \\ F(X_{i})\\ \vdots \\ F(X_{N}) \end{array}\right]}_{N\times 1}.$$

Let *F* represent a vector of objective function values, $F_{i}$ denote assessed value of objective function for *i*-th FL. These objective function values serve as key metrics for assessing the quality of CSs (individuals in the population). Specifically, the best objective function value corresponds to the optimal individual, while the worst value corresponds to the least optimal individual. As positions of FLs are updated during each iteration of FLOA, new values of the objective function are computed. Subsequently, the position of the best individual must be updated in every iteration. At the end of FLOA, the best solution determined during iterations is chosen as the final solution to the optimization problem.

#### 3.6.2. Mathematical Modelling of FLOA

In every iteration of FLOA, updating the location of FL is carried out in two different stages: Exploration and Exploitation. The Exploration stage mimics an FL’s hunting behavior as it roams in search of prey. Its purpose is to spread the search across the SS, probing diverse regions to uncover new, potentially optimal areas. In the Exploitation stage, after feeding, the FL climbs to a high vantage point. This is analogous to the algorithm concentrating on the most promising solutions identified earlier. By refining these select regions, this phase improves the best candidates and drives convergence toward the global optimum.

- **Phase 1: Hunting (Exploration)** A key behavioral trait of FL is its unique hunting method. The FL remains still until it spots potential prey before attacking. In the context of optimization algorithms, simulating FL’s movement toward its prey leads to significant shifts in the locations of members within SS, thereby improving the algorithm’s capability to explore and search globally. During the first stage of FLOA, the locations of members in SS are updated depending on FL’s hunting behavior.
  In FLOA design, each FL considers the position of other individuals in the population with improved objective function values as potential prey. The positions of the Candidate Prey (CP) for each FL are determined by using Equation (18):
  (18) $${\mathrm{CP}}_{i}=\left\{X_{k}:F_{k}<F_{i},\;k\neq i\right\},$$
  where, i=1,2,…,Nandk∈{1,2,…,N}. ${\mathrm{CP}}_{i}$ represents CP for the *i*-th FL, $X_{k}$ denotes member in population with value of objective function better than *i*-th FL and $F_{k}$ denotes value of objective function. In FLOA, FL randomly chooses a CP and launches an attack. Depending on the modeled motion of FL toward the selected prey, a new location for every individual is computed by using Equation (19). If the location offers an improved value of objective function, the former location of the individual is replaced by the new one as shown in Equation (20).
  (19) $${x_{i}^{d}}^{\left(P_{1}\right)}=x_{i}^{d}+r\cdot \left(SP_{i}^{d}-I\cdot x_{i}^{d}\right),$$
  (20) $$X_{i}=\left\{\begin{array}{cc} X_{i}^{\left(P_{1}\right)} & \mathrm{if}\;F_{i}^{\left(P_{1}\right)}<F_{i}\\ X_{i} & \mathrm{otherwise} \end{array}\right.,$$
  where, $X_{i}^{\left(P_{1}\right)}$ represents new recommended position of *i*-th FL based on initial phase of FLOA, ${x_{i}^{d}}^{\left(P_{1}\right)}$ denotes its *d*-th dimension, $F_{i}^{\left(P_{1}\right)}$ signifies value of objective function, *r* denotes random number which is normally distributed within the interval [0,1], $SP_{i}^{d}$ represents *d*-th dimension of chosen prey for *i*-th FL, *I* denotes a number at random from the range {1,2}, *N* represents number of FLs and *m* represents number of decision variables.
- **Phase 2: Climbing the Tree (Exploitation)** Once feeding is done, the FL retreats to the top of a nearby tree. Simulation of movement to the tree top leads to small adjustments in the locations of members in the population within SS, thus, enhancing the algorithm’s capability to perform local search. In the second stage of FLOA, the locations of members in SS are updated based on FL’s strategy of retreating to the tree top after feeding. By modeling the FL’s movement toward the tree, a new location for every individual is determined by using Equation (21). If the new location offers an improved value of objective function, it replaces the former location of the respective individual as specified in Equation (22).
  (21) $${x_{i}^{d}}^{\left(P_{2}\right)}=x_{i}^{d}+\left(1-2r\right)\cdot \frac{\left(ub_{d}-lb_{d}\right)}{t},$$
  (22) $$X_{i}=\left\{\begin{array}{cc} X_{i}^{\left(P_{2}\right)} & \mathrm{if}\;F_{i}^{\left(P_{2}\right)}<F_{i}\\ X_{i} & \mathrm{otherwise} \end{array}\right.,$$
  where, $X_{i}^{\left(P_{2}\right)}$ represents new recommended location of *i*-th FL depending on second phase of FLOA, ${x_{i}^{d}}^{\left(P_{2}\right)}$ denotes *d*-th dimension, $F_{i}^{\left(P_{2}\right)}$ signifies value of objective function, *t* denotes iteration counter and *T* denotes maximum number of iterations in FLOA. Pseudocode for the implementation of FLOA, which provides a structured overview of its operations, is outlined in Algorithm 1.

**Algorithm 1:** Frilled Lizard Optimization Algorithm (FLOA)

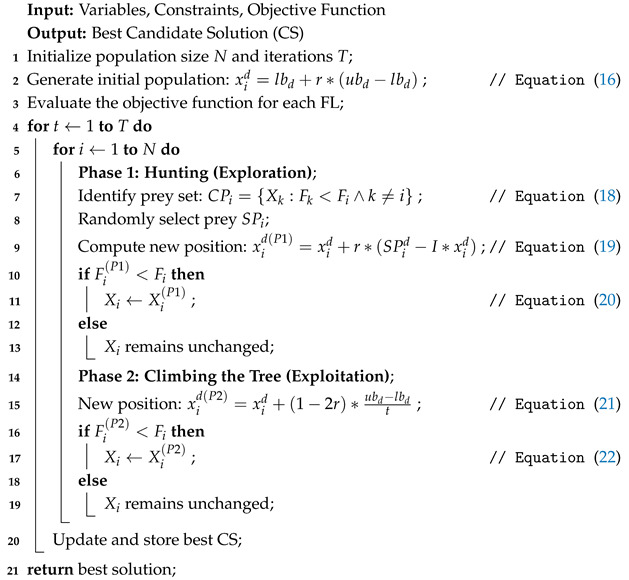



### 3.7. Security and Cryptographic Mechanisms

To ensure secure and efficient data aggregation, we integrate cryptographic mechanisms including bilinear pairings, fully homomorphic encryption (FHE), the Chinese Remainder Theorem (CRT), and online/offline signatures. These techniques protect data confidentiality, integrity, and authentication while enabling privacy-preserving computations [23,24,25].

#### 3.7.1. Bilinear Pairing Setting

Two multiplicative cyclic groups, *G* and $G_{T}$, are formed with a prime order *p* and a generator g∈G. A bilinear map is defined as $e:G\times G\to G_{T}$, which satisfies the following properties:

- **Bilinear:** For any u,v∈G and any $a,b\in Z_{p}^{*}$, it holds that $e\left(u^{a},v^{b}\right)=e{\left(u,v\right)}^{ab}$.
- **Non-degenerate:** The generator *g* of *G* must satisfy $e\left(g,g\right)\neq 1_{G_{T}}$.
- **Computable:** There exists an efficient algorithm to compute e(u,v) for all u,v∈G.

To establish the security foundation for SSDBFAN, the following hard problem is considered:

**q-Strong Diffie-Hellman (q-SDH) Problem:** Let *G* be a cyclic group of prime order *p* with generator *g*, and let *x* be a random element in $Z_{p}^{*}$. Given (q+1)-tuple $\left(g,g^{x},g^{x^{2}},\ldots ,g^{x^{q}}\right)$, the challenge is to compute a pair $\left(m,g^{1/(x+m)}\right)$ for some $m\in Z_{p}^{*}$. The q-SDH problem is said to be (q,t,ε)-hard if no adversary A running in time *t* can solve it with success probability at least ε.

(23) $$\mathrm{Pr}\left[A\left(g,g^{x},g^{x^{2}},\ldots ,g^{x^{q}}\right)=\left(m,g^{1/(x+m)}\right)\;\mathrm{for}\;\mathrm{some}\;m\in Z_{p}^{*}\right]<\varepsilon .$$

 **Theorem 1.** Under the (q,t,ε)-SDH assumption, the probability that a t-time algorithm solves the q-SDH problem in G is less than ε. This probability accounts for the random selection of $x\in Z_{p}^{*}$ and internal randomness used by the adversary A [24].

#### 3.7.2. Fully Homomorphic Encryption Scheme (FHES) from Lattices

To ensure data confidentiality during aggregation, the proposed scheme employs post-quantum cryptography via a homomorphic encryption system based on lattices [25]. This system allows computation over encrypted data, enabling secure aggregation without revealing individual values.

Let us define the convolution polynomial ring *R* as:

(24) $$R=Z\left[x\right]/\langle x^{N}-1\rangle .$$

Here, *R* denotes the set of polynomials β(x)∈Z[x] reduced modulo $x^{N}-1$. Any polynomial a(x) in *R* must have integer coefficients and a degree strictly less than *N*. Polynomials of degree ≥N are reduced by substituting $x^{N}$ with 1.

Thus, the ring *R* includes all polynomials of the form:

(25) $$a\left(x\right)=a_{0}+a_{1}x+\ldots +a_{N-1}x^{N-1}\;\mathrm{where}\;a_{i}\in Z.$$

Every such element can also be treated as a vector in Euclidean space $Z^{N}$ by interpreting the coefficients of the polynomial as vector components. For example, a(x) maps to the vector $\left(a_{0},a_{1},\ldots ,a_{N-1}\right)$. The FHES consists of the following three main operations:

1. **Key Generation (KeyGen)**: Similar to NTRU [26], the scheme operates on the ring Z[x] with a polynomial of degree *N*, denoted $f\left(x\right)=x^{N}-1$. To instantiate the encryption mechanism, two lattices *I* and *J* are constructed in Z[x]. Lattice *I* is assigned a good basis $B_{I}$, while *J* has both a secret (bad) basis $B_{J}^{sk}$ and a public (good) basis $B_{J}^{pk}$. These lattices are required to be “prime” in the sense that any vector in $Z^{N}$ can be written as the sum of a vector from *I* and a vector from *J*, analogous to the idea of coprime integers.
  The public key is $\left(B_{J}^{pk},B_{I}\right)$, and the secret key is $\left(B_{J}^{sk},B_{I}\right)$.
2. **Encryption (ENC)**: To encrypt a message *m*, the encryption algorithm proceeds as follows:
  - Randomly choose a vector $v\in Z^{N}$
  - Compute: $T^{\prime }=B_{I}v+m$
  - Encrypt by reducing modulo $B_{J}^{pk}$:
    (26) $$T=\left(B_{I}v+m\right)\;\mathrm{mod}\;B_{J}^{pk}.$$
  The resulting ciphertext *T* lies in the coset space defined by $B_{J}^{pk}$.
3. **Decryption (DEC)**: To decrypt the ciphertext *T*, the original message *m* is recovered using:
  (27) $$m=\left(T\;\mathrm{mod}\;B_{J}^{sk}\right)\;\mathrm{mod}\;B_{I}.$$
  This effectively extracts the message embedded in the encrypted vector using the known secret bases.
4. **Computational Complexity and Feasibility of FHE:** It is a known fact that Fully Homomorphic Encryption (FHE) schemes involve considerable computational complexity in terms of polynomial multiplication and modular reduction in high-dimensional space, which is based on lattice operations. Typically, such operations are known to vary from O(n log n) to higher complexities depending on the parameters. To make the proposed framework feasible in FANETs, the proposed framework is designed to restrict FHE operations to aggregation-related operations, which are typically performed by Cluster Heads (CHs) and Ground Control Stations (GCSs), where such operations are likely to be performed with considerable computational capabilities compared to individual UAV nodes, which are responsible for performing light-weight cryptographic operations such as encryption and signature generation, where online/offline signatures help reduce computational complexity.

#### 3.7.3. Chinese Remainder Theorem (CRT)

The concept of relatively prime moduli in CRT [27] supports the development of efficient data aggregation mechanisms at the Cluster Head (CH) level within the FANET network. This theorem guarantees the existence of a unique solution for systems of linear congruences, allowing data originating from multiple FANET devices (even with heterogeneous formats) to be effectively aggregated at the Ground Control Station (GCS).

 **Theorem 2.** *Let $r_{1},r_{2},\ldots ,r_{m}$ be positive integers that are pairwise relatively prime, and let $s_{1},s_{2},\ldots ,s_{m}$ be arbitrary integers. Then, the system of congruences:*

(28) $$c\equiv s_{i}\;\mathrm{mod}\;r_{i}\;\mathrm{for}\;1\leq i\leq m,$$

*has a unique solution modulo $R=r_{1}\cdot r_{2}\cdot \ldots \cdot r_{m}$, given by:*

(29) $$x\equiv s_{1}R_{1}y_{1}+s_{2}R_{2}y_{2}+\cdots +s_{m}R_{m}y_{m}\;\mathrm{mod}\;R,$$

*where $R_{i}=\frac{R}{r_{i}}$ and $y_{i}\equiv R_{i}^{-1}\;\mathrm{mod}\;r_{i}$ for 1≤i≤m.*

#### 3.7.4. Online/Offline Signatures

The online/offline signature mechanism enhances secure communication by dividing the signing process into pre-computable (offline) and real-time (online) phases. This is efficiently implemented using the Hash–Sign–Switch Paradigm (HSSP) [14], defined over a large prime $p_{1}$ and generator $g_{1}\in G_{p_{1}}$.

Trapdoor keys $c,d\in Z_{p_{1}}^{*}$ determine the function:

(30) $$H_{\mathrm{hssp}}\left(f,j,k\right)=g_{1}^{f}\cdot g_{2}^{j}\cdot g_{3}^{k},\;\mathrm{where}\;g_{2}=g_{1}^{c},\;g_{3}=g_{1}^{d}.$$

The HSSP function offers the following properties:

- **Computability:** Efficiently computable using a polynomial-time algorithm.
- **Collision Resistance:** Infeasible to find distinct triples mapping to the same hash without trapdoor keys.
- **Trapdoor Collision:** Controlled collisions can be generated using the private keys.

Based on HSSP, the online/offline signature scheme includes:

- **Setup:** Generates the key pair $\left(Sigkey_{sk},VK_{pk}\right)$ using a security parameter $1^{\psi }$.
- **Signature-offline:** Precomputes $\Sigma_{\mathrm{offline}}$ and state data $SA_{t}$ using $Sigkey_{sk}$.
- **Verification-offline:** Validates $\Sigma_{\mathrm{offline}}$ using $VK_{pk}$.
- **Signature-online:** Computes $\Sigma_{\mathrm{online}}$ using $Sigkey_{sk},SA_{t}$, and message *m*.
- **Verification-online:** Uses $VK_{pk},m,\Sigma_{\mathrm{offline}},\Sigma_{\mathrm{online}}$ to verify message authenticity. The final signature is $\Sigma =(\Sigma_{\mathrm{offline}},\Sigma_{\mathrm{online}})$.

This dual-phase signature significantly reduces real-time computation, making it ideal for resource-constrained UAV environments in SSDBFAN.

### 3.8. SSDBFAN Protocol Description

This section introduces the operational workflow of the proposed SSDBFAN (Scalable and Secure Cluster-Based Data Aggregation with Blockchain for Flying Ad Hoc Networks). It details the sequential phases of the protocol, including system setup, secure registration, encrypted report generation, aggregation, response dissemination, and blockchain integration. Each phase is designed to ensure confidentiality, authentication, integrity, and scalability within dynamic FANET environments. Table 2 contains the notations and their descriptions, which are essential for analyzing and discussing the proposed scheme.

**Table 2:** Symbol description table.

Symbol	Description	Symbol	Description
TA	Trusted Authority	$er_{i}$	Encryption done by CM side
GCS	Ground Control Station	$u_{i}^{\prime }$	Online signature
$\left(B_{J}^{pk},B_{I}\right)$	TA’s public key	$VK_{online}$	Online signature verification
$\left(B_{J}^{sk},B_{I}\right)$	Trusted authority’s private key	$AGGR_{cp}$	Aggregate encrypted messages by CH
A	Adversary	$Q_{j}$	Private key of CH
p	Largest prime number	$AD_{pt}$	Aggregated plain text
$H_{hssp}$	Hash–Sign–Switch Paradigm function	$ID_{j}$	ID of CH
c, d	Two trapdoor keys	$\Delta_{t}$	Timestamp
$\Psi_{pub}$	Set of public parameters	$AGP_{x}$	Aggregated data in blockchain
M_key	Master private key	$ub_{num}$	Unique block number
$Sigkey_{prk}$	Signature private key	$H_{prev}$	Previous block
$VK_{puk}$	Verification public key	MerkleRoot	Fingerprint of entire Merkle Tree
$s_{i}$	Blinding factor	$U_{R_{j}}$	Extracting insights from the blockchain
CH, CM	Cluster Head and Cluster Member	$\Sigma_{offline}$	Offline signature token
$TS_{i}$	Timestamp of CM ‘i’	$1^{\psi }$	Security parameter
$autk_{i}$	Authorized user key	$VK_{pk}$	Verification key
$\Sigma_{BLS-SF}$	Boneh–Lynn–Shacham signature method	$VK_{Aggr}$	Aggregation signature public key
$TG_{i}^{offline}$	Offline tag	$Recov_{Aggr}$	Recovered aggregated report

#### 3.8.1. The System Setup

Upon receiving a request for data aggregation from GCSs, TA employs FHES from Lattices to produce public as well as private key pair $\left(\left(B_{J}^{pk},B_{I}\right),\left(B_{J}^{sk},B_{I}\right)\right)$. To guarantee security, TA randomly selects *s* and $s_{1}$ as security factors and a bilinear map $e:G\times G\to G_{T}$ of prime order $\left(p_{1}\right)$ with $|p_{1}|=s_{1}$. TA also defines three one-way hash functions,

(31) $$H_{0}:{\left\{0,1\right\}}^{*}\to G,\;H_{1}:{\left\{0,1\right\}}^{*}\to Z_{p_{1}}^{*},\;H_{2}:G\to Z_{p_{1}}^{*},$$

and HSSP function

(32) $$H_{hssp}:Z_{p_{1}}^{*}\to G.$$

Additionally, TA selects three arbitrary elements $\tilde{\sigma },\tilde{x}\in Z_{p_{1}}^{*},Q\in G$ and computes $e{\left(g_{1},g_{1}\right)}^{\tilde{\sigma }}$ and $\tilde{P}=g_{1}^{\tilde{x}}$. There are δ FANET devices during the aggregation request period. TA publicly publishes parameters as given by:

(33) $$\Psi_{\mathrm{pub}}=\left\{p_{1},B_{I},G,G_{T},e,g_{1},\delta ,\tilde{P},Q,e{\left(g_{1},g_{1}\right)}^{\tilde{\sigma }},H_{0},H_{1},H_{2},H_{hssp}\right\},$$

while keeping master private keys confidential and forwarding to GCSs:

(34) $$M_{\mathrm{key}}=\left(B_{J}^{pk},B_{J}^{sk},\tilde{\sigma },\tilde{x}\right).$$

#### 3.8.2. Registration

The registration phase enables each Cluster Member ($CM_{i}$) to securely join the SSDBFAN system and obtain authentication credentials from the (GCSs). This phase establishes the cryptographic identity of $CM_{i}$ while preserving privacy through a blinded identity construction.

**Figure 2:**
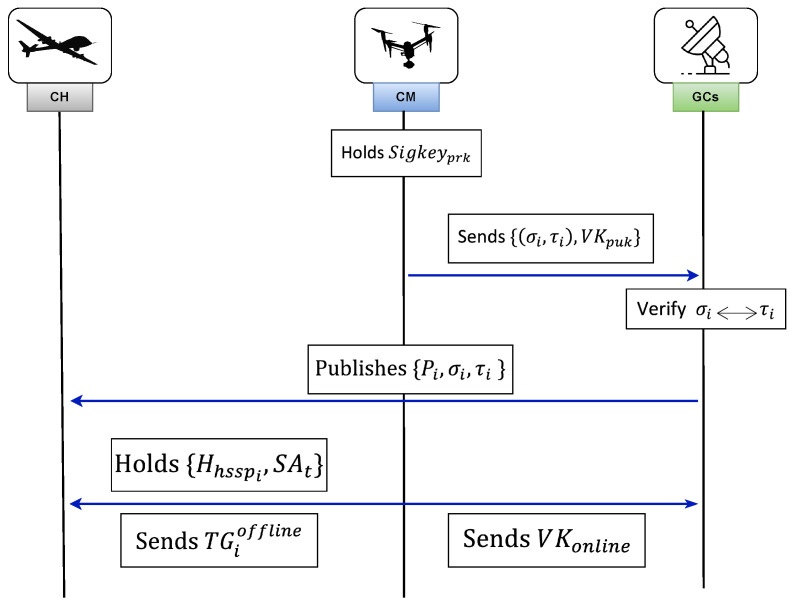
Registration and data transmission process.

1. User Registration: To initiate registration, $CM_{i}$ selects a random value $Q_{i}\in Z_{\left(p_{1}\right)}^{*}$ and computes its corresponding public key:
  (35) $$P_{i}=g_{1}^{Q_{i}}.$$
  The pair $\left(Q_{i},P_{i}\right)$ forms the private–public signing key pair $\left({\mathrm{Sigkey}}_{\mathrm{prk}},{\mathrm{VK}}_{\mathrm{puk}}\right)=\left(Q_{i},P_{i}\right)$. The secrecy of $Q_{i}$ ensures that only $CM_{i}$ can generate valid signatures associated with $P_{i}$.
  To protect identity information during transmission, $CM_{i}$ selects a random blinding factor $s_{i}$ and computes:
  (36) $$f_{i}=H_{1}({\mathrm{ID}}_{i}\|{\mathrm{TS}}_{i}\left\|s_{i}\right),$$
  where ${\mathrm{ID}}_{i}$ denotes the identity and ${\mathrm{TS}}_{i}$ denotes the timestamp.
  Next, $CM_{i}$ constructs the registration tuple:
  (37) $$\begin{array}{cc} \sigma_{i} & =g_{1}^{f_{i}}, \end{array}$$
  (38) $$\begin{array}{cc} \tau_{i} & =f_{i}-Q_{i}H_{2}\left(\sigma_{i}\right). \end{array}$$
  The tuple $\left(\sigma_{i},\tau_{i}\right)$ together with $P_{i}$ is transmitted to the GCSs via the Cluster Head, as shown in Figure 2.
2. Authentication: Upon receiving the registration request, GCSs verify the legitimacy of $CM_{i}$ by checking:
  (39) $$\sigma_{i}=g_{1}^{\tau_{i}}P_{i}^{H_{2}\left(\sigma_{i}\right)}.$$

If the equation holds, the registration request is accepted, and GCSs generate the corresponding authentication credentials for $CM_{i}$. These credentials are later used for offline signature generation and secure report submission.

#### 3.8.3. Generation of Reports

**Figure 3:**
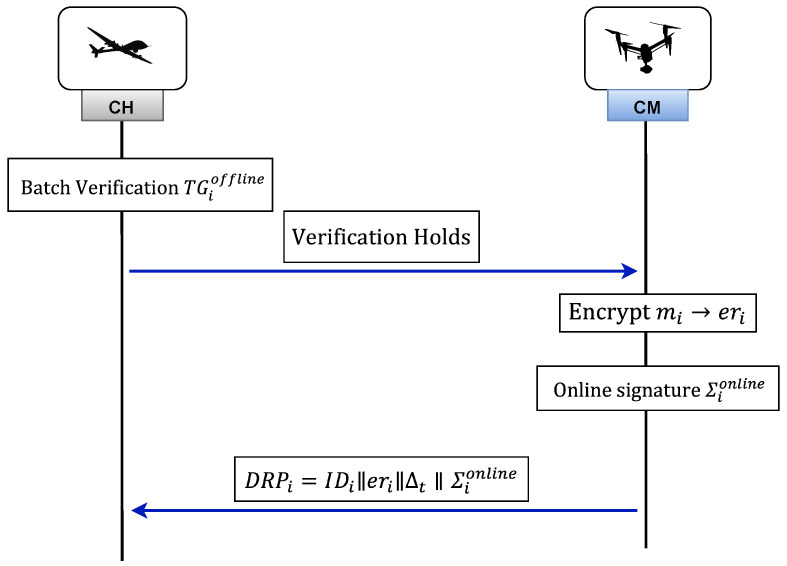
Encrypted report generation and transmission process.

Once $CM_{i}$ receives the offline tag $TG_{i}^{offline}$ from CH, the CH performs offline verification. After successful verification of the offline signature, CH proceeds to receive the encrypted data from $CM_{i}$ along with the corresponding online signature. Figure 3 illustrates the encrypted report generation and transmission process.

1. **Verifying Offline Signatures:**
  When CH receives offline tags from $CM_{i}\;(1\leq i\leq \delta )$, it verifies the signatures using the verification public key $VK_{puk}$ by checking
  (40) $$e\left(g_{1},\Sigma_{i}^{BLS-SF}\right)=e\left(P_{i},H_{0}\left(H_{hssp_{i}}\right)\right).$$
  Batch verification is performed as
  (41) $$\prod_{i=1}^{\delta }e\left(P_{i},H_{0}\left(H_{hssp_{i}}\right)\right)=e\left(g_{1},\prod_{i=1}^{\delta }\Sigma_{i}^{BLS-SF}\right).$$
  If valid, the algorithm returns “accept”; otherwise, “reject”.
2. **Encrypting Data:** After successful offline verification, $CM_{i}$ encrypts sensed data $m_{i}$ using homomorphic encryption:
  (42) $$er_{i}=g_{1}^{m_{i}}\cdot v_{i}\;\mathrm{mod}\;B_{I},$$
  where $v_{i}$ is randomly selected from $Z^{N}$.
3. **Generating Online Signature:** The online signature is generated based on state information $SA_{i}=\left(f_{i},j_{i},k_{i}\right)$ as
  (43) $$u_{i}^{\prime }=\left(\left(f_{i}-er_{i}\right)+\left(j_{i}-j_{i}^{\prime }\right)y+k_{i}z\right)z^{-1},$$
  where $j_{i}^{\prime }\in Z_{\left(p_{1}\right)}^{*}$ and $\Sigma_{i}^{online}=\left(j_{i}^{\prime },k_{i}^{\prime }\right)$. Finally, $CM_{i}$ forwards
  (44) $$DRP_{i}=\{ID_{i}\|er_{i}\|\Delta_{t}\left\|\Sigma_{i}^{online}\right\},$$
  to the neighboring CH, where $\Delta_{t}$ denotes the current timestamp.

#### 3.8.4. Aggregation

**Figure 4:**
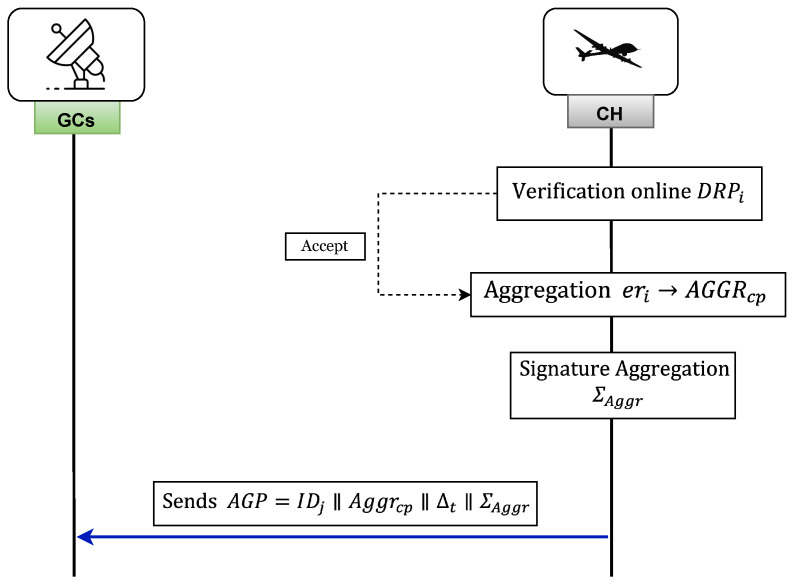
Aggregation process.

The process of report aggregation begins with CH receiving the reports $DRP_{i}$, 1≤i≤δ, from CM. These reports are then subjected to an online verification algorithm in order to determine the validity of $\Sigma_{i}^{online}$. Figure 4 provides an in-depth overview of the aggregation phase.

1. **Online Signature Verification:** Upon receiving $\Sigma_{i}^{online}$, CH first checks the timestamp $\Delta_{t}$ and verifies its validity using the verification function $VK_{online}$. This involves checking whether
  (45) $$H_{hssp}\left(f_{i},j_{i},k_{i}\right)=H_{hssp}\left(er_{i},j_{i}^{\prime },k_{i}^{\prime }\right)$$
  is valid or not. If the equation is valid, the result will be “accept”; if not, the result will be “reject.”
2. **Report Aggregation:** After verifying the online signature, CH moves on to calculate the combined encrypted value as
  (46) $$AGGR_{cp}=\prod_{i=1}^{\delta }\left(er_{i}\;\mathrm{mod}\;B_{I}\right).$$
3. **Signature Generation for Aggregation:** To generate aggregation signature, CH randomly selects a private key $Q_{j}\in Z_{\left(p_{1}\right)}^{*}$, where $ID_{j}$ is the identity of cluster head $CH_{j}$. This aggregation signature, denoted as $\Sigma_{Aggr}$, is calculated as
  (47) $$\Sigma_{Aggr}={\left(H_{0}(ID_{j}\|AGGR_{cp}\left\|\Delta_{t}\right)\right)}^{Q_{j}}.$$
  Finally, $CH_{j}$ sends the aggregated report to the GCs.
  (48) $$AGP_{x}=\{ID_{j}\|AGGR_{cp}\|\Delta_{t}\left\|\Sigma_{Aggr}\right\}$$

#### 3.8.5. Reading Reports

**Figure 5:**
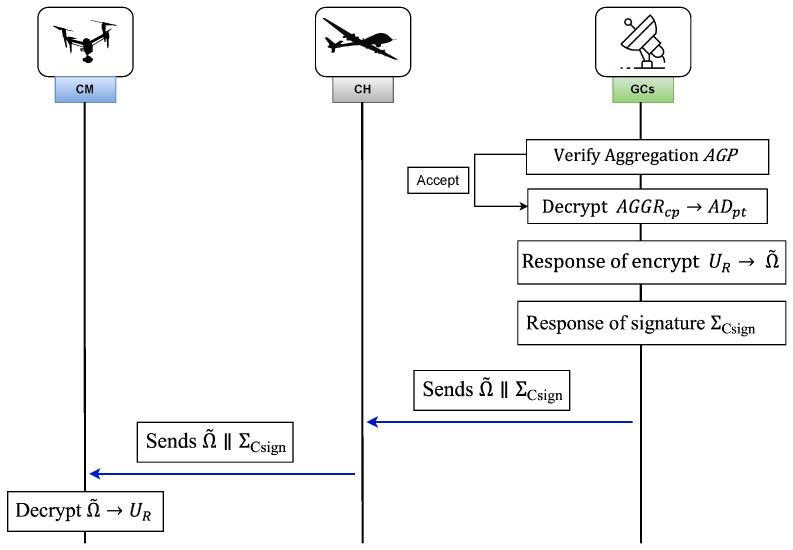
Report reading and response process.

For reading the aggregated outcome obtained from GCSs, $CH_{j}$ follows a particular set of steps and forwards respective response to CM. Figure 5 illustrates the overview of reading reports and response stage. First, GCSs validate aggregation signature $\Sigma_{Aggr}$ from received data report by using the following equation.

(49) $$e\left(g_{1},\Sigma_{Aggr}\right)=e(P_{j},H_{0}(ID_{j}\|AGGR_{cp}\|\Delta_{t})),$$

where $P_{j}=g_{1}^{Q_{j}}$. In case of successful verification, the algorithm returns “accept”, otherwise returns “reject”. Upon verifying the aggregation signature, GCSs convert the aggregated encrypted $AGGR_{cp}$ as shown below.

(50) $$AGGR_{cp}=\prod_{i=1}^{\delta }\left(er_{i}\;\mathrm{mod}\;B_{I}\right)=g_{1}^{AD_{pt}}\cdot \prod_{i=1}^{\delta }\left(v_{i}\;\mathrm{mod}\;B_{I}\right).$$

Aggregated plaintext ($AD_{pt}$) may be decrypted easily by GCSs as it uses fully homomorphic encryption (FHE). The encrypted data is transformed and aggregated plaintext is obtained as shown below.

(51) $$AD_{pt}=\sum_{i=1}^{\delta }AD_{pt_{i}}.$$

#### 3.8.6. Response

Following assessment of the combined plaintext ($AD_{pt}$), GCSs send a response $U_{R}\in G_{T}$ to CH within the coverage zone. To ensure confidentiality of response, it is sent as an encrypted message. The following steps are involved.

**Step 1:** GCSs select $\tilde{\tau }\in Z_{\left(p_{1}\right)}^{*}$ which is a random number and then compute $\tilde{\Omega }=\left({\tilde{er}}_{1},{\tilde{er}}_{2},{\tilde{er}}_{3}\right)$, where

(52) $${\tilde{er}}_{1}=M_{R}\cdot e{\left(g_{1},g_{1}\right)}^{\tilde{\sigma }\tilde{\tau }}\;\mathrm{mod}\;n,\;{\tilde{er}}_{2}=g_{1}^{\tilde{\tau }},\;{\tilde{er}}_{3}={\left(C/O\right)}^{\tilde{\tau }}.$$

The GCSs then generate a signature

(53) $$\Sigma_{Csign}={\left(H_{0}\left(\tilde{\Omega }\|\Delta_{\Omega }\right)\right)}^{x},$$

using present time stamp $\Delta_{\Omega }$ and send $\tilde{\Omega }\|\Sigma_{Csign}$ to CH which covers a set of CMs.

**Step 2:** Upon receiving $\tilde{\Omega }\|\Sigma_{Csign}$, the CH verifies authenticity of $\tilde{\Omega }$ by ensuring

(54) $$e\left(g_{1},\Sigma_{Csign}\right)=e\left(C,H_{0}\left(\tilde{\Omega }\|\Delta_{\Omega }\right)\right).$$

If it holds, CH broadcasts $\tilde{\Omega }$ within the coverage area.

**Step 3:** Upon receiving $\tilde{\Omega }$ which is verified by CH, every user $\delta_{i}$ in set δ uses authorized key

(55) $$autk_{i}=\left(g_{1}^{\tilde{\sigma }}\cdot C^{t_{i}},O^{t_{i}},g_{1}^{t_{i}}\right),$$

for decrypting data set $U_{R}$.

(56) $$\frac{e({\tilde{er}}_{2},g_{1}^{\tilde{\sigma }}\cdot C^{t_{i}})}{e\left({\tilde{er}}_{2},Q^{t_{i}}\right)e\left({\tilde{er}}_{3},g_{1}^{t_{i}}\right)}=e{\left(g_{1},g_{1}\right)}^{\tilde{\alpha }\tilde{\beta }}.$$

(57) $$\frac{{\tilde{er}}_{1}}{e{\left(g_{1},g_{1}\right)}^{\tilde{\sigma }\tilde{\tau }}}=\frac{U_{R}\cdot e{\left(g_{1},g_{1}\right)}^{\tilde{\sigma }\tilde{\tau }}}{e{\left(g_{1},g_{1}\right)}^{\tilde{\sigma }\tilde{\tau }}}=U_{R}.$$

The recovered information $U_{R,\delta_{i}}$ is employed for making dynamic intelligent decisions, while ensuring privacy.

#### 3.8.7. Adding Data to the Blockchain

GCSs are responsible for integrating aggregated data ($AGP_{x}$) into the blockchain by constructing a new block, consisting of a block header and a block body. The block header includes a unique block number, a reference to the previous block’s hash, a timestamp $\Delta_{t}$, the aggregated data $AGP_{x}$, a SHA3-256 hash for integrity verification, and a Merkle Root. The block header hash is computed as:

(58) $$H_{\mathrm{current}}=\mathrm{SHA}3\text{-}256\left(ub_{\mathrm{num}}+H_{\mathrm{prev}}+\Delta_{t}+AGP_{x}+\mathrm{MerkleRoot}\right).$$

**Figure 6:**
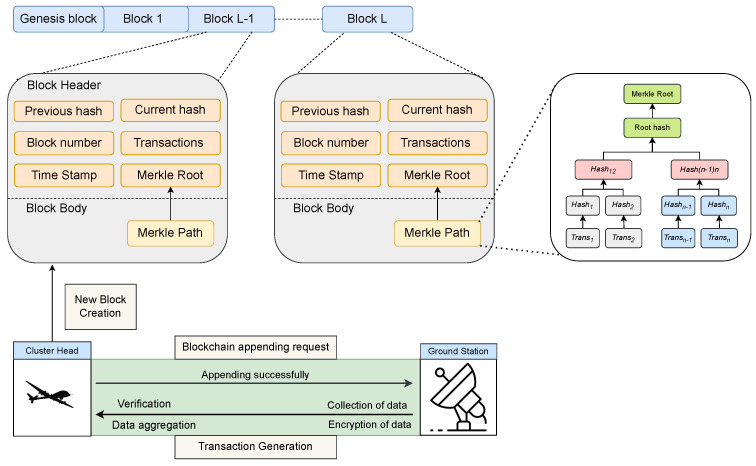
Blockchain construction and Merkle root generation process in the SSDBFAN framework.

The block body contains transaction records within a given time frame, structured into a Merkle Tree, where the Merkle Root ensures tamper resistance. Once created, the block is broadcast to the network, validated by participating nodes, and added to their blockchain copies if consensus is reached. Upon confirmation by the majority, CH notifies all nodes, ensuring the block is permanently linked to the existing chain. Figure 6 provides an overview of the blockchain and Merkle root generation process.

#### 3.8.8. Extracting Insights from the Blockchain

GCSs, as the trusted entity, access the blockchain to retrieve aggregated data ($AGP_{x}$) stored in blocks. Leveraging the Chinese Remainder Theorem, GCSs reconstruct the complete dataset $U=(U_{1},U_{2},\ldots ,U_{k})$ from aggregated values without exposing individual user data, ensuring privacy preservation while enabling meaningful insights. The decryption process is computed as:

(59) $$U_{R_{j}}=\left(\sum_{i=1}^{n}\sum_{j=1}^{k}\left(AGP_{ij}\;\mathrm{mod}\;Q\right)\right)\;\mathrm{mod}\;B_{I}=\sum_{i=1}^{n}AGP_{ij}.$$

This final step enables GCSs to extract aggregated statistics while preserving individual confidentiality, completing the privacy-preserving data aggregation workflow.

## 4. Security Analysis

An overall analysis of security properties and models, along with design objectives, is presented. The main focus is on confidentiality, authentication, privacy, integrity, and unforgeability.

### 4.1. Authentication

In the proposed SSDBFAN mechanism, an authentication mechanism in the registration stage based on an improved Schnorr’s signature scheme is embedded [28], which is secure based on the discrete logarithm problem. The accuracy of authentication is given by:

(60) $$g_{1}^{\beta_{i}}\cdot Y_{i}^{H_{2}\left(\alpha_{i}\right)}=g_{1}^{\left(r_{i}-X_{i}\cdot H_{2}\left(\alpha_{i}\right)\right)}\cdot g_{1}^{X_{i}\cdot H_{2}\left(\alpha_{i}\right)}=g_{1}^{r_{i}}=\alpha_{i}.$$

Specifically, an attacker cannot falsify registration knowledge $\left\{\alpha_{i},\beta_{i}\right\}$ without obtaining the real identity ($ID_{i}$) of $SD_{i}$ as $ID_{i}$ is secured using a one-way hash function ($H_{1}$) and preserved secretly. Furthermore, even if an attacker steals $ID_{i}$ of $SD_{i}$, it cannot obtain the value of hash function ($r_{i}$), as $r_{i}$ is concealed using a blinding factor ($k_{i}$) that is randomly chosen, thus, guaranteeing security of the private key ($X_{i}$) of the signature. Hence, authentication between SD and CC is secure.

### 4.2. Confidentiality and Privacy-Preservation

In the proposed SSDBFAN mechanism, fully homomorphic encryption (FHE) is used for encrypting sensed data as well as aggregating ciphertext based on the homomorphic property. Confidentiality and privacy of data may be assured through the following three aspects:

- During report generation, private data $\left(m_{i}\right)$ of $D_{i}$ is encrypted as $c_{i}=Enc\left(m_{i}\right)$, which represents the ciphertext under the adopted homomorphic encryption scheme. As the scheme is secure against Chosen Plaintext Attack (CPA), there are no chances for the information to be disclosed.
- During report aggregation, ES will not be able to recover the plaintext of individuals without the secret key. It aggregates obtained ciphertexts as $c=Enc\left(\sum_{i=1}^{\omega }m_{i}\right)$ based on the homomorphic property. This ensures that data confidentiality and privacy are preserved even when ES cannot be trusted.
- Assume that an external attacker who eavesdrops on the complete communication channel between SD and CC exists. Even if individual ($c_{i}$) and aggregated (*c*) ciphertexts along with aggregated plaintext (*m*) are available, individual plaintext ($m_{i}$) cannot be obtained due to the semantic security of the encryption scheme. Confidentiality and privacy of sensitive data of $D_{i}$ can be secured.

### 4.3. Unforgeability and Integrity

An online or offline signature scheme is included for ensuring data integrity while reducing computation costs. The scheme is unforgeable under EU-CMA (Existential Unforgeability under Chosen Message Attack), thus, ensuring data integrity. Without querying the signing oracle token on $m^{*}\in Z_{p_{1}}^{*}$, adversary A will not be able to forge $\left(m^{*},\Sigma^{*}\right)$ to guarantee signature $\left(\Sigma^{*}\right)$ validity using private key $\left({\mathrm{Sig}}_{sk}\left(\right)\right)$ in polynomial time. The problem is represented as a theorem.

 **Theorem 3.** *A signature system is $\left(t,k_{1},k_{2},\epsilon \right)$ protected from EU-CMA if q-SDH may be resolved using algorithm B with significant probability $\epsilon^{\prime }\geq \epsilon /3-k_{2}/p$ in polynomial time.*

**Proof.**  A contradiction scheme is used for proving this theorem, assuming that A queries offline as well as online signature oracle for $k_{1}$ and $k_{2}$ number of times correspondingly on $m_{i}$, $k_{2}=k\leq q_{1}$. Let $\left(\Sigma_{\mathrm{off}},\Sigma_{\mathrm{on}}\right)$ represent full signatures of real signing oracle following $k_{2}$ queries by A. Legitimate forgery signature $\left(\Sigma_{\mathrm{off}}^{*},\Sigma_{\mathrm{on}}^{*}\right)$ is returned by A on fresh message $m^{*}$ with probability ϵ. Algorithm B produces $\left(g,g^{\tau },g^{\tau^{2}},\ldots ,g^{\tau^{q}}\right)$ which represents q-SDH occurrence that focuses on building a fresh signature $\left(\Sigma_{\mathrm{off}}^{*},\Sigma_{\mathrm{on}}^{*}\right)$ and effectively handling the q-SDH problem. A performs attacks that fall into the following cases:

- **Case 1:** $g^{m^{*}}g_{2}^{s^{*}}g_{3}^{u^{*}}\neq g^{m_{i}}g_{2}^{s_{i}}g_{3}^{u_{i}}$ for all $i\in \{1,\cdots ,k_{2}\}$.
- **Case 2:** $g^{m^{*}}g_{2}^{s^{*}}g_{3}^{u^{*}}=g^{m_{i}}g_{2}^{s_{i}}g_{3}^{u_{i}}$, $i\in \left\{1,\cdots ,k_{2}\right\},s^{*}\neq s_{i}$.
- **Case 3:** $g^{m^{*}}g_{2}^{s^{*}}g_{3}^{u^{*}}=g^{m_{i}}g_{2}^{s_{i}}g_{3}^{u_{i}}$, $i\in \left\{1,\cdots ,k_{2}\right\},s^{*}=s_{i},u^{*}\neq u_{i}$.

**CASE 1:**

*Initialization:* B randomly selects numbers $c,d\in Z_{p}^{*}$. It obtains the private key of the signature, SK=(a,c,d). It sends public key for verification to A: $VK_{\mathrm{online}}=\left(g,g_{1},g_{2},g_{3}\right)$, $g_{1}=g^{a},g_{2}=g^{c},g_{3}=g^{d}$.

*Signature offline Queries:* If the *i*-th offline query is considered by A, $1\leq i\leq q_{1}$, then B returns the *i*-th offline signature to A: $\Sigma_{i}^{\mathrm{off}}=\left(H_{0}{\left(H_{ch_{i}}\right)}^{a},{\bar{H}}_{ch_{i}}\right)$. $H_{ch_{i}}=g^{r_{i}}\cdot g_{2}^{s_{i}}\cdot g_{3}^{u_{i}}=g^{\left(r_{i}+s_{i}\cdot y+z\right)}$, $\left(f_{i},i_{i},j_{i}\right)\in Z_{p}^{*}$. B stores the above values. Actually, $\Sigma_{i}^{\mathrm{off}}$ is valid as $e\left(g,H_{0}{\left(H_{{\mathrm{hssp}}_{i}}\right)}^{a}\right)=e\left(g_{1},H_{0}\left(H_{{\mathrm{hssp}}_{i}}\right)\right)$.

Use $c_{i}=f_{i}+i_{i}\cdot c+j_{i}\cdot d$.

*Signature online Queries:* A considers the *i*-th online query, $1\leq i\leq q_{2}$. B returns $\Sigma_{i}^{\mathrm{on}}=\left(i_{i}^{\prime },j_{i}^{\prime }\right)$ as the *i*-th online signature to A: $u_{i}^{\prime }=\frac{\left(f_{i}-m_{i}\right)+\left(i_{i}-i_{i}^{\prime }\right)c+j_{i}\cdot d}{d}$, where $i_{i}^{\prime }\in Z_{p}^{*}$

Validity of $\Sigma_{i}^{\mathrm{on}}$ is assured by $H_{{\mathrm{hssp}}_{i}}\left(f_{i},i_{i},j_{i}\right)=H_{ch_{i}}\left(m_{i},i_{i}^{\prime },j_{i}^{\prime }\right)$.

*Forgery:* Finally, A sends a legal signature forgery message $\left(m^{*},i^{*},j^{*},i_{*}^{\prime },j_{*}^{\prime }\right)$ fulfilling Case 1. As: $g^{m^{*}}g_{2}^{j^{*}}g_{3}^{j^{*}}\neq g^{m_{i}}g_{2}^{i_{i}}g_{3}^{j_{i}}$, so $c^{*}=m^{*}+i^{*}\cdot c+j^{*}\cdot d\neq c_{i}$.

Algorithm B exists for solving the q-SDH problem with a likelihood not less than ϵ/3 (Similar to Case 1).

The difference between the cases lies in the phases: in Case 1, B forges the value of Hash–Sign–Switch Paradigm (HSSP) function $\left(H_{\mathrm{hssp}}^{*}\right)$, whereas trapdoors ’c’ and ’d’ are forged in other cases. Hence, only forging is focused in the remaining cases.

**CASE 2:**

*Forgery:* B forges trapdoor ‘y’, and the private key of signature is given by SK=(x,a,d). The probability of this case is at least ϵ/3. $s^{*}=s_{i}$ happens with likelihood 1/p as $s_{i}$ is randomly chosen from $Z_{p}^{*}$. For the complete game, $s^{*}=s_{i}$ happens with likelihood $q_{2}/p$. Once A offers fake signature $\left(m^{*},\Sigma_{\mathrm{off}}^{*}\left(a,f^{*},i^{*},j^{*}\right),\Sigma_{\mathrm{on}}^{*}\left(i_{*}^{\prime },j_{*}^{\prime }\right)\right)$ that satisfies case 2, B may effectively determine: $a=c=\frac{\left(m^{*}-m_{i}\right)+\left(j^{*}-j_{i}\right)z}{i_{i}-i^{*}}$,

B succeeds with likelihood $\epsilon /3-q_{2}/p$ for handling the q-SDH problem.

**CASE 3:**

*Forgery:* B forges trapdoor ‘d’ and assigns conforming private key =(x,c,a). The likelihood of $j^{*}=j_{i}$ is given by $q_{2}/p$. B solves the q-SDH problem with likelihood $\epsilon /3-q_{2}/p$ by determining $a=d=\frac{\left(m^{*}-m_{i}\right)+\left(i^{*}-i_{i}\right)d}{j_{i}-j^{*}}$ in polynomial time for ’i’. A forges valid signature $\left(m^{*},\Sigma_{\mathrm{off}}^{*}\left(a,f^{*},i^{*},j^{*}\right),\Sigma_{\mathrm{on}}^{*}\left(i_{*}^{\prime },j_{*}^{\prime }\right)\right)$ that satisfies Case 3. B solves the q-SDH problem with likelihood $\epsilon /3-q_{2}/p$ in polynomial time. The theorem is proven due to contradictions between the reasoning outcome and q-SDH assumption. □

### 4.4. Runtime Overhead of Cryptographic Operations

The runtime overhead of cryptographic operations is factored into the simulation by taking into account the processing delay for the operations performed during the process of encryption, signature computation, and aggregation. Lightweight operations such as online signatures have a minimal delay, whereas heavier operations such as homomorphic aggregation have a higher delay at the CH and GCS levels. This is to avoid the overload of the UAV nodes and thereby keep the communication real-time and feasible.

## 5. Performance Evaluation

This section outlines the simulation setup, the criteria used to evaluate performance, and the resulting findings from the simulation. The implementation includes actual libraries for cryptographic functions, such as NFL, a library for lattice-based cryptographic functions; PBC, a library for bilinear pairing-based functions; OpenSSL, a library for hashing and other standard cryptographic functions; and Crypto++, a library for auxiliary functions related to cryptographic calculations. These libraries are integrated with NS-3 [29], to perform actual cryptographic functions, and the time required for these functions is also accounted for during the simulation, represented by actual processing delays. Blockchain implementation has been carried out at the application layer, and actual functions such as block generation, hashing, and validation are carried out using OpenSSL, while message passing has been implemented using RapidJSON [30], a lightweight JSON parser and generator, to reduce the load on UAV nodes, with cluster heads handling blockchain functions. Experiments are carried out under similar conditions to compare different approaches and the simulation parameters utilized are detailed in Table 3. The performance results presented in this section support the design advantages summarized in Table 1, particularly in terms of clustering efficiency, scalability, and system performance. It should be noted that certain aspects, such as security and blockchain-related properties, are evaluated analytically rather than through direct simulation.

### 5.1. Simulation Environment and Evaluation Framework

The performance of the proposed SSDBFAN framework is evaluated through a comprehensive set of metrics designed to assess computational efficiency, communication performance, network stability, optimization effectiveness, and blockchain scalability. These metrics are selected to reflect the key challenges in FANET environments, including limited computational resources, dynamic topology, and the need for secure and efficient data aggregation. Specifically, computational cost and delay are analyzed to evaluate the impact of cryptographic operations on UAV nodes. Communication overhead and throughput are examined to measure network efficiency under increasing node density. Cluster lifetime and processing time are considered to assess network stability and system responsiveness. Furthermore, the effectiveness of the FLOA-based clustering mechanism is evaluated through comparative analysis with existing optimization algorithms. Finally, blockchain-related metrics are analyzed to examine the scalability and overhead introduced by the distributed ledger component.

The performance evaluation is conducted under varying network sizes to analyze the scalability and robustness of the proposed SSDBFAN framework. The number of UAV nodes is gradually increased to observe its impact on computational cost, communication efficiency, network stability, and blockchain performance. Comparative analysis is performed against existing schemes, namely BBMDA [35] and BBKMS [7], under identical simulation conditions to ensure fairness. The corresponding results are presented through graphical analysis in the following subsections.

### 5.2. Computational Cost and Average Delay Analysis

The report generation process for $CM_{i}$ in the SSDBFAN scheme involves two exponentiations in the group $Z_{n^{2}}$ to generate the ciphertext $c_{i}$ and three multiplications in group G to determine the online signature ($\Sigma_{\mathrm{online}}$). During report aggregation, the cluster head $CH_{j}$ verifies the online signatures and aggregates the gathered ciphertexts. This step requires three exponentiations in G and δ multiplications in $Z_{n^{2}}$. It is important to note that hash operations and multiplications in $Z_{n^{2}}$ are considered negligible in contrast to exponentiation and pairing operations.

**Figure 7:**
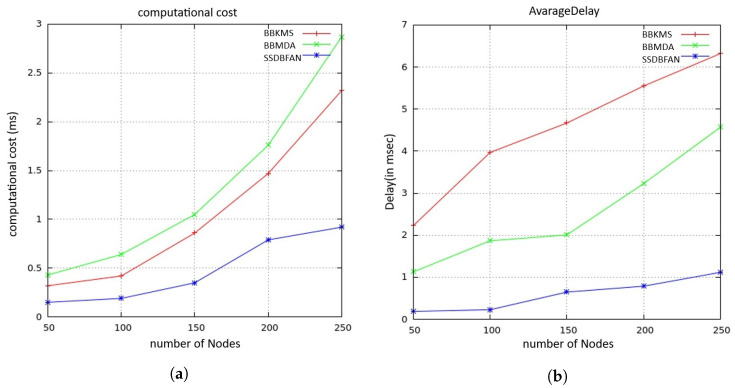
Comparison of computational cost and average delay for different protocols. (**a**) Number of nodes vs. computational cost. (**b**) Number of nodes vs. average delay.

Finally, $CH_{j}$ performs a single exponentiation in G to generate the aggregation signature ($\Sigma_{\mathrm{Aggr}}$). Upon receiving the aggregated report from $CH_{j}$, the ground Control station (GCS) verifies $\Sigma_{\mathrm{Aggr}}$ and decrypts the aggregated ciphertext $c_{i}$ to obtain the plaintext sum. This decryption process involves two pairing operations and two exponentiations in $Z_{n^{2}}$. This analysis reveals that the SSDBFAN scheme minimizes the number of time-consuming cryptographic operations required on the $CM_{i}$ device, particularly in relation to signature generation. Figure 7a illustrates a comparison of overall computational costs across 3 different schemes. As the figure shows, the proposed SSDBFAN mechanism offers substantial efficiency advantages in terms of computational costs compared to the other two referenced mechanisms. This efficiency is achieved by shifting a major portion of the complex operations to the offline phase.

Figure 7b shows the relationship between number of nodes and the average delay. SSDBFAN aims to reduce data transmission delays by streamlining the data aggregation process and improving overall communication efficiency. It leverages sophisticated data handling methods alongside optimized routing strategies to ensure faster and more reliable delivery of aggregated data. These improvements lead to lower latency and enhanced performance across the network, offering clear advantages over conventional methods like BBKMS and BBMDA. With its focus on swift data delivery and reduced communication lag, SSDBFAN is suitable for real-time applications that demand low-latency responses.

### 5.3. Communication Efficiency Analysis

This subsection evaluates the communication efficiency of the proposed SSDBFAN framework in terms of communication overhead and throughput. These metrics are essential for assessing network performance in FANET environments, where bandwidth limitations and dynamic topology significantly impact system efficiency. Communication overhead represents the total amount of data transmitted within the network, including both control packets and data packets. It directly affects bandwidth utilization and energy consumption, making it a critical performance metric in UAV networks.

**Figure 8:**
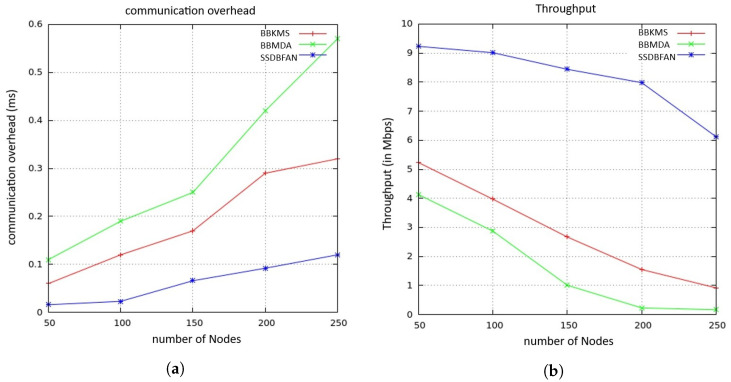
Comparison of communications overhead and average throughput for different protocols. (**a**) No. of nodes vs. communications overhead. (**b**) No. of nodes vs. average throughput.

Figure 8a presents an analysis of communication overhead. The plotted graph depicts communication overhead (in milliseconds) as a function of the number of nodes for three schemes: BBKMS, BBMDA, and SSDBFAN. The SSDBFAN framework utilizes a single communication phase, consolidating the key steps typically found in a two-phase approach. This node-to-node communication phase enables each cluster member to transmit its data report, denoted as $DRP_{i}=\left\{CM_{ij},c_{i},\Delta_{t},\Sigma_{i}^{\mathrm{online}}\right\}$, directly to a designated aggregator or control center.

For secure communication cost evaluation, a lattice-based Fully Homomorphic Encryption Scheme (FHES) is employed to ensure confidentiality during aggregation. This post-quantum cryptography technique enables encrypted data operations without revealing individual inputs. Assuming a total of *n* users in the FANET during a given period, the total communication overhead is calculated as:

(61) $$STS=\sum_{i=1}^{n}SSD_{i}$$

The graph shows that SSDBFAN consistently achieves the lowest communication overhead, followed by BBMDA and then BBKMS. As the number of nodes increases, BBKMS exhibits a linear rise in overhead, BBMDA remains moderate, and SSDBFAN maintains minimal growth. This highlights SSDBFAN’s communication efficiency and scalability, making it well-suited for large-scale FANET deployments.

Throughput is defined as the rate of successful data delivery over the network within a given time interval and reflects the overall effectiveness of data transmission.

Figure 8b shows the correlation between number of nodes and average throughput. The SSDBFAN approach optimizes data transmission efficiency by reducing delays and enhancing network throughput. SSDBFAN achieves improved performance through a dynamic scheduling mechanism that adjusts in real-time to changing network conditions, promoting consistent and timely data delivery. Its design also emphasizes energy-efficient data transmission, contributing to enhanced throughput and reduced latency across the network. By optimizing routing and transmission processes, SSDBFAN delivers better overall efficiency compared to traditional methods such as BBKMS and BBMDA.

### 5.4. Network Stability and Processing Efficiency

This subsection evaluates the performance of the proposed SSDBFAN framework in terms of cluster lifetime and transaction processing time. These metrics are essential for analyzing network stability and system responsiveness in dynamic FANET environments. Cluster lifetime is defined as the duration for which a cluster remains stable before re-clustering is required. It reflects the effectiveness of the cluster-head selection mechanism and directly impacts network stability and control overhead. A longer cluster lifetime reduces the frequency of re-clustering operations, thereby lowering communication overhead.

**Figure 9:**
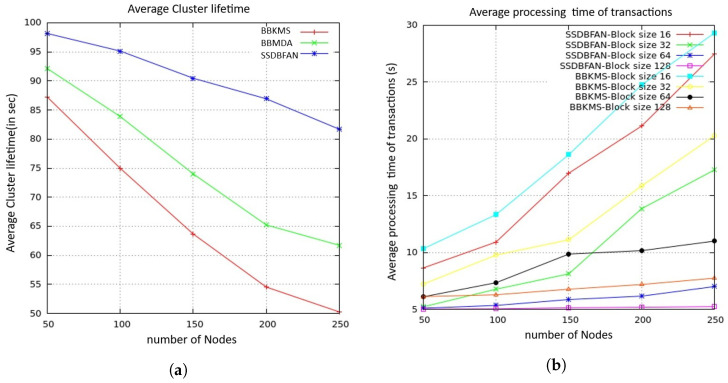
Comparison of cluster lifetime and transaction processing time for different protocols. (**a**) Average cluster lifetime. (**b**) Average processing time of transactions.

In Figure 9a, average cluster lifetime (in seconds) is shown against number of nodes for three different protocols: SSDBFAN, BBMDA, and BBKMS. For SSDBFAN, average cluster lifetime decreases steadily as the number of nodes increases from 40 to 200, starting at around 65 s and dropping to about 35 s. BBMDA exhibits a similar trend but with slightly better performance than SSDBFAN, beginning at about 60 s and reducing to around 30 s. In contrast, BBKMS shows a significantly higher average cluster lifetime compared to the other two protocols, starting at around 95 s and maintaining above 80 s as the number of nodes increases to 200. This graph highlights that BBKMS offers a much longer cluster lifetime compared to SSDBFAN and BBMDA, This is because BBKMS emphasizes maintaining cluster persistence by reducing the frequency of cluster reconfiguration. In contrast, SSDBFAN adopts a more adaptive clustering strategy based on the FLOA optimization algorithm, which considers multiple factors such as residual energy, trust, and mobility. While this adaptive approach may lead to slightly shorter cluster lifetimes, it results in better load balancing, improved reliability, and enhanced resistance to malicious or unstable nodes.

Transaction processing time represents the delay required to validate and store data within the blockchain layer. This metric is critical for evaluating the feasibility of integrating blockchain mechanisms into time-sensitive FANET applications.

Figure 9b illustrates the processing time, measured in seconds, for different block sizes: 16, 32, 64, and 128 bytes across varying numbers of nodes. The figure highlights the average transaction processing time corresponding to these block sizes and node configurations. As the number of nodes increases, the transaction processing time also rises. When smaller block sizes are used with a higher number of nodes within a given interval, the number of transactions tends to exceed the block’s handling capacity. Conversely, increasing block size results in greater communication overhead. Despite these challenges, the proposed scheme demonstrates a lower average processing time compared to existing approaches and ensures faster transaction handling while maintaining data integrity and security, making it suitable for real-time and large-scale FANET deployments.

### 5.5. Optimization Algorithm Impact (FLOA)

The experiments in the previous subsections were conducted with up to 250 UAV nodes to evaluate baseline performance metrics such as computational cost, communication efficiency, and network stability under moderate network conditions. However, to further assess the scalability and robustness of the proposed SSDBFAN framework, additional experiments are conducted with an extended network size of up to 500 nodes.

This extended evaluation is particularly important for analyzing the behavior of the FLOA-based clustering mechanism and the blockchain layer under large-scale network conditions, where challenges such as increased communication overhead, consensus delay, and storage requirements become more significant. By increasing the network size, the proposed framework is evaluated under more demanding scenarios to validate its scalability and security effectiveness in dense FANET deployments.

Furthermore, to demonstrate the effectiveness of the proposed FLOA-based clustering mechanism, a comparative analysis is conducted with representative optimization algorithms, including OPA [36], HBA [37], and RSA [38]. These algorithms are selected due to their diverse optimization strategies and their relevance in network optimization problems. Specifically, OPA represents a probabilistic-based optimization approach, HBA models adaptive foraging behavior for dynamic environments, and RSA is inspired by cooperative and exploration–exploitation balancing mechanisms. Comparing with these methods provides a comprehensive evaluation across different optimization paradigms. The performance of the proposed FLOA-based clustering approach is evaluated using three key metrics: cluster lifetime, average delay, and throughput. Cluster lifetime reflects the stability of the clustering structure, average delay represents the time required for data transmission, and throughput indicates the rate of successful data delivery in the network.

**Figure 10:**
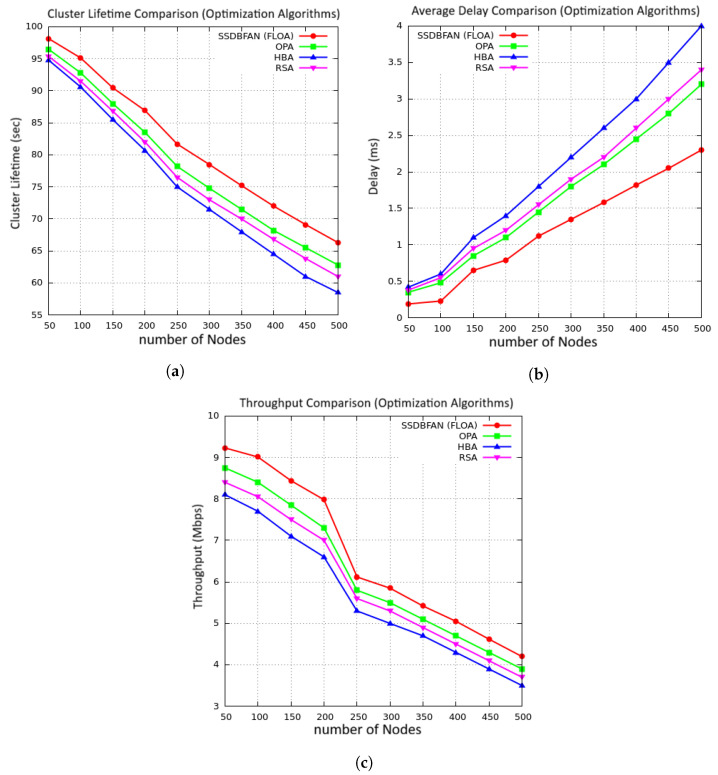
Comparison of cluster lifetime, average delay, and throughput for different optimization algorithms. (**a**) Cluster lifetime comparison. (**b**) Average delay comparison. (**c**) Throughput comparison.

Figure 10a, presents the comparison of the average cluster lifetime using different optimization techniques. With an increase in the number of nodes, the cluster lifetime is reduced for all optimization techniques due to energy consumption. However, the proposed framework has shown better performance in terms of cluster lifetime compared to OPA [36], HBA [37], and RSA [38] techniques. This is due to the FLOA-based CH selection mechanism, where energy, mobility, and trust are considered to provide balanced energy usage in the FANET. The performance of OPA and RSA is moderate, while the performance of HBA is reduced with an increase in nodes due to inefficient CH selection. Figure 10b shows the average delay performance of different optimization algorithms. From the figure, it is noted that the delay is increasing with the number of UAV nodes due to high communication overhead. However, the proposed SSDBFAN framework is performing better with less delay compared to OPA, HBA, and RSA in all scenarios. From the figure, it is noted that the delay is reduced due to optimal CH selection. Although OPA and RSA are performing better with moderate delay, HBA is performing with high delay due to inefficient cluster formation. These observations indicate that FLOA is performing better with more efficient and stable cluster formation. In Figure 10c, the comparison of the throughput using the proposed SSDBFAN optimization method, based on the FLOA optimization algorithm, and other optimization techniques, namely, OPA, HBA, and RSA, is presented, considering the changing sizes of the network. From the results, as the number of UAV nodes is increased, the throughput value decreases in all optimization techniques, owing to increased congestion in the network. However, the throughput value is high in the proposed optimization method, irrespective of the density of the nodes in the network. This is because the FLOA optimization algorithm is efficient in the selection of the cluster head (CH), which is responsible for the data transmission. The other optimization techniques, namely, OPA and RSA, have moderate reduction in the throughput value, while the HBA optimization technique is found to have less throughput value compared to the other techniques.

### 5.6. Blockchain Scalability Analysis

**Figure 11:**
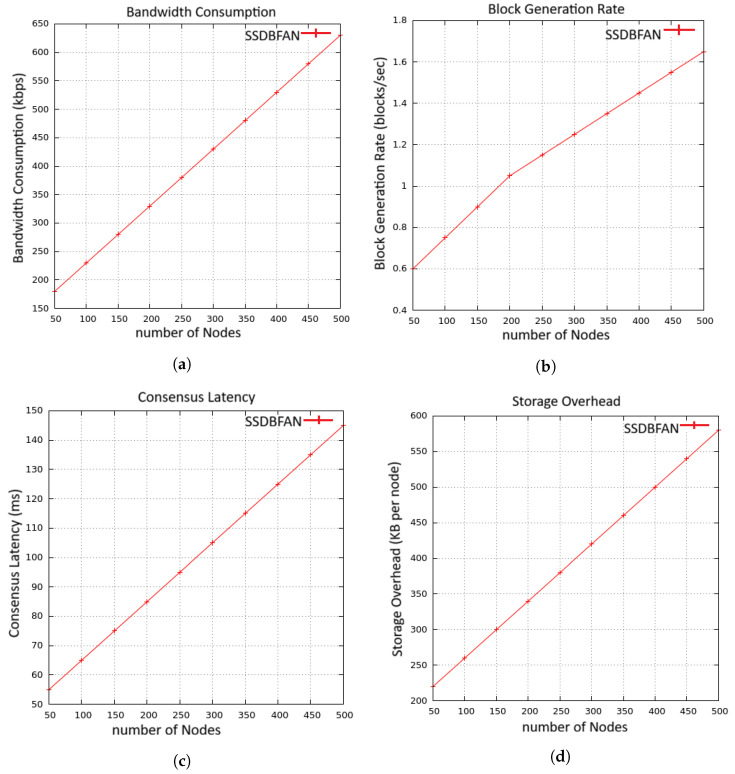
Comparison of bandwidth consumption, block generation rate, consensus latency, and storage overhead for the SSDBFAN blockchain layer. (**a**) Number of nodes vs bandwidth consumption. (**b**) Number of nodes vs block generation rate. (**c**) Number of nodes vs consensus latency. (**d**) Number of nodes vs storage overhead.

The blockchain scalability of the proposed SSDBFAN framework is evaluated using four key metrics: bandwidth consumption, block generation rate, consensus latency, and storage overhead. These metrics collectively reflect the communication cost, processing efficiency, and scalability of the blockchain layer under large-scale network conditions. Bandwidth consumption represents the amount of data exchanged during blockchain operations. Figure 11a, shows the bandwidth consumption due to blockchain synchronization with an increase in the number of UAV nodes. From the figure, it is clear that the bandwidth consumption is increasing almost linearly with an increase in the number of nodes. This is obvious, as more nodes will result in more blocks being propagated in the network. In the proposed framework of SSDBFAN, the blockchain communication is limited to the cluster heads. Therefore, the bandwidth consumption is under control. Hence, the increase is moderate, indicating that the framework is capable of handling more nodes without any congestion in the network.

Block generation rate indicates how frequently new blocks are created. Figure 11b, depicts the block generation rate with respect to the number of UAV nodes. With an increase in the number of nodes in the network, more transactions are added to the blockchain, thus, increasing the block generation rate gradually. In the proposed framework, namely SSDBFAN, the block generation rate is kept at an optimal level. This is done to avoid generating more blocks, which could lead to congestion in the blockchain.

Consensus latency refers to the time required to validate and confirm a block. Figure 11c shows the consensus latency with varying sizes of the network. The latency is found to increase with the number of nodes, as the communication delay and complexity are high in the process of validating the blocks. Despite the increase in latency, the system shows linear growth, unlike exponential growth, as achieved through the cluster-based consensus mechanism, where only the participating nodes are the CHs, thereby reducing the complexity. The results show that the system maintains acceptable latency levels even in the case of large networks.

Storage overhead represents the memory required to store blockchain data. Figure 11d, shows the storage overhead per node required to maintain the blockchain data. The storage requirement is expected to increase as the number of nodes is increased, as the number of blocks is accumulated over time. In the proposed SSDBFAN, the storage overhead is optimized by only using the aggregated data, as opposed to the raw data collected by the UAV. The linear growth trend shows that the system can scale well without imposing an excessive storage burden on the UAV nodes. Overall, the proposed SSDBFAN framework reduces blockchain overhead while maintaining efficient and secure data validation, demonstrating its suitability for scalable and security-aware FANET environments.

## 6. Conclusions and Future Directions

This paper introduces SSDBFAN, a scalable and secure cluster-based data aggregation framework for Flying Ad Hoc Networks (FANETs). The proposed approach integrates the Frilled Lizard Optimization Algorithm (FLOA) for efficient cluster head selection with blockchain technology and post-quantum cryptographic techniques, including lattice-based homomorphic encryption and the Chinese Remainder Theorem, to ensure privacy-preserving data aggregation. Additionally, a hybrid online/offline signature mechanism is employed to achieve secure and efficient authentication with reduced computational overhead. The performance of the proposed framework is evaluated using NS-3 simulations under varying network sizes. Experimental results demonstrate that SSDBFAN significantly improves communication efficiency, reduces computational cost, and enhances network stability compared to existing schemes. Furthermore, scalability analysis with up to 500 UAV nodes confirms that the proposed framework effectively controls blockchain overhead, including bandwidth consumption, consensus latency, and storage requirements. Comparative evaluation with existing optimization algorithms shows that FLOA achieves superior performance in terms of cluster stability, delay, and throughput. These results validate the effectiveness of SSDBFAN as a scalable and security-aware solution for large-scale FANET environments.

## Figures and Tables

**Table 1:** Qualitative comparison of existing FANET security and clustering approaches.

Reference	Key Idea	Advantages	Limitations
[15]	Blockchain-based group key agreement for UAV networks	Enhances security and decentralization; ensures transparent key agreement	Limited clustering support; lacks optimization mechanism
[16]	Secure communication framework for UAV networks	Provides secure key management and authentication	Limited scalability and clustering efficiency
[4]	Blockchain-assisted UAV security mechanism	Improves trust and security in UAV communication	No clustering or optimization support
[17]	Secure routing and communication scheme	Enhances scalability and communication efficiency	Limited key update flexibility
[18]	UAV communication security framework	Supports efficient data exchange and scalability	Lacks clustering and advanced optimization
[19]	Lightweight blockchain-based UAV system	Improves efficiency and reduces overhead	Partial clustering support; limited security depth
[20]	Secure UAV communication model	Provides reliable key management	No clustering or advanced privacy mechanisms

**Table 3:** Experimental simulation parameters.

Parameters	Definitions
Simulator	NS 3.25
Simulation area (width × depth)	1000 × 1000 m
Number of users	50, −250, −500
Number of ground stations	1
Mobility model	Random Waypoint
Mac Layer	IEEE 802.11b
Packet size	512 bytes
Modules used	Antenna, config store, mobility, flow monitor, and internet
External libraries used	NFL library [31], PBC library [32], OpenSSL [33], Crypto++ [34]
Hash Algorithm	SHA-256
Routing protocol	SSDBFAN
Transmission range	250 m
Simulation Time	300 s

## Data Availability

The original contributions presented in this study are included in the article. Further inquiries can be directed to the corresponding author.
